# Heterogeneity and efficacy of antipsychotic treatment for schizophrenia with or without treatment resistance: a meta-analysis

**DOI:** 10.1038/s41386-019-0577-3

**Published:** 2019-11-25

**Authors:** Yuya Mizuno, Robert A. McCutcheon, Stefan P. Brugger, Oliver D. Howes

**Affiliations:** 1 Department of Psychosis Studies, Institute of Psychiatry, Psychology & Neuroscience, King’s College London, London, SE5 8AF UK; 2 Department of Neuropsychiatry, Keio University School of Medicine, Tokyo, Japan; 3 Psychiatric Imaging Group, MRC London Institute of Medical Sciences, Hammersmith Hospital, London, UK; 4 Institute of Clinical Sciences, Faculty of Medicine, Imperial College London, London, UK; 5 Centre for Academic Mental Health, University of Bristol, Bristol, UK; 6 Division of Psychiatry, University College London, London, UK; 7 Cardiff University Brain Research Imaging Centre, School of Psychology, Cardiff University, Cardiff, UK

**Keywords:** Outcomes research, Pharmacology

## Abstract

Two important clinical questions are whether there is a subtype of schizophrenia which responds differently to clozapine relative to other antipsychotics, and whether greater efficacy of clozapine is dependent on the degree of treatment-resistance. The authors address this by examining both variability and magnitude of response in patients treated with clozapine and other antipsychotics for both treatment-resistant schizophrenia (TRS) and non-resistant schizophrenia. Double-blind randomised controlled trials comparing clozapine with other antipsychotics in patients with schizophrenia were identified using five databases. Standard deviations and means of change in total, positive, and negative symptoms were extracted. Variability ratio (VR) and coefficient of variation ratio (CVR) were used to quantify relative variability in symptom change. Hedges’ *g* was used to quantify mean differences. Ten TRS studies (*n* = 822) and 29 non-TRS studies (*n* = 2566) were meta-analysed. Relative variability in change of total symptoms did not differ significantly between clozapine and other antipsychotics in TRS studies (VR = 1.84; 95%CI, 0.85–4.02). These findings were similar with CVR, and for positive and negative symptoms. Clozapine was superior to other antipsychotics in improving total symptoms in both TRS (*g* = 0.34; 95%CI, 0.13–0.56) and non-TRS (*g* = 0.20; 95%CI, 0.08–0.32) studies. Furthermore, clozapine was superior in improving positive symptoms in both study groups, but not for negative symptoms. Pooled effect sizes showed no significant difference between TRS and non-TRS studies. These findings do not support a subtype of schizophrenia which responds specifically to clozapine. Clozapine is more effective than other antipsychotics irrespective of treatment-resistance, arguing for its use more generally in schizophrenia. PROSPERO CRD42018086507

## Introduction

Schizophrenia is a severe mental illness with a lifetime prevalence of approximately 0.7% [1]. It is characterised by psychotic symptoms, including delusions and hallucinations, negative symptoms including, amotivation and social withdrawal, and cognitive impairment. Antipsychotic drugs remain the cornerstone of treatment for schizophrenia [2, 3]. However, there is heterogeneity in how patients respond to antipsychotics from the early stages of illness [4, 5]. About one-third of patients have treatment-resistant schizophrenia (TRS) [6], defined as inadequate response to two or more trials of first-line (non-clozapine) antipsychotic treatment at adequate dose and duration [7]. Treatment-resistance is a major challenge to clinical management, and is associated with high medical costs and increased disability relative to schizophrenia in general [8].

A landmark clinical trial in 1988 established clozapine as superior to other antipsychotics in treating TRS [9]. For over two decades since then, clozapine has had a unique position as the only medication licensed for patients with TRS [10]. Furthermore, clozapine treatment is associated with reduced hospitalisations [11] and long-term mortality [12] relative to other antipsychotics. However, a recent network meta-analysis of therapy for TRS utilising both direct and indirect comparisons between antipsychotics showed no difference between clozapine and most other antipsychotics [13]. This has challenged clozapine’s unique position in the therapeutic arsenal for schizophrenia.

Subsequently, a systematic review by the Treatment Response and Resistance in Psychosis (TRRIP) working group found that in 50% of clinical trials of antipsychotics for TRS it was unclear how TRS was established [7]. Furthermore, a number of clinical trials included patients who were intolerant rather than treatment-resistant to previous antipsychotic treatment [7]. Heterogeneity in study populations due to variability in inclusion criteria and/or the inclusion of patients with treatment-intolerance as well as TRS may obscure the superiority of a therapy for TRS. Recent network [13] and conventional [14] meta-analyses comparing antipsychotics in TRS have selected studies based on broad definitions of treatment-resistance, later accounting for study characteristics in sensitivity analyses. However, no meta-analyses have compared the efficacy of clozapine between studies of strictly-defined TRS and studies of schizophrenia not exclusive for treatment-resistance. Furthermore, none have examined the association between the rigour in definitions of TRS based on the TRRIP consensus criteria [7] and clozapine’s efficacy. These investigations are crucial in establishing clozapine’s efficacy in treatment-resistant illness.

If treatment is specifically effective for a condition, in addition to showing a greater improvement, it should also show a more homogenous response relative to comparator drugs that are not effective (e.g. TRS patients treated with clozapine show consistent improvement, whereas those receiving other antipsychotics respond more variably). Alternatively, if treatment is specifically effective for a subgroup of patients with the condition, it will result in a more variable response compared to medications that are not effective (e.g., a subgroup of TRS patients improve significantly with clozapine, whereas those receiving other antipsychotics show a more consistent pattern of minimal change). This can be determined by measuring the variability of response. Nakagawa et al. proposed a novel method to meta-analyse variability [15], which we recently used to compare variability between regional brain structure [16] and drug-placebo response [17] in schizophrenia.

**Fig. 1:**
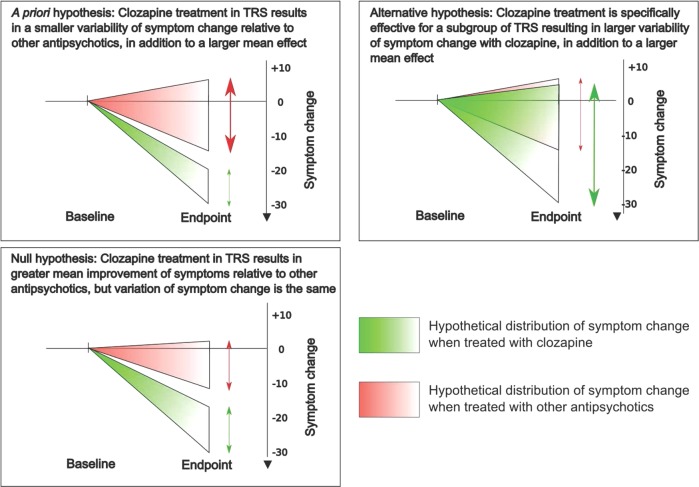
Hypothesised result for the meta-analysis of variance in patients with strictly-defined treatment-resistant schizophrenia (TRS). The trajectory of symptom change for TRS patients treated with clozapine and other antipsychotics are illustrated in green and red, respectively. The vertical arrows represent the pooled variability of symptom change within each treatment group.

**Fig. 2:**
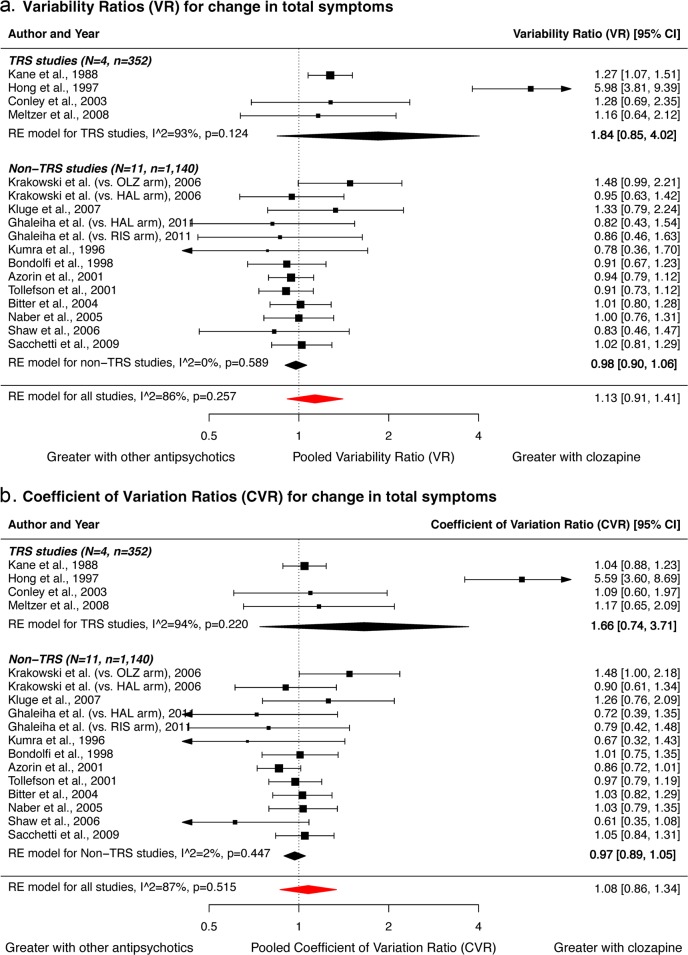
Forest plot showing variability ratios (VR) and coefficient of variation ratios (CVR) for change in total symptoms in studies of clozapine relative to other antipsychotics for the treatment of patients with strictly-defined treatment-resistant schizophrenia (TRS) and other non-refractory schizophrenia (non-TRS). In studies of TRS, there is no significant alteration in the summary variability ratio (VR = 1.84, *p* = 0.124), indicating that the variability in response to treatment is the same in patients receiving clozapine as other antipsychotics. Furthermore, there is no significant alteration in the summary coefficient of variation ratio (CVR = 1.66, *p* = 0.220), indicating that the variability in response to treatment is the same in patients receiving clozapine as other antipsychotic drugs after adjusting for greater symptomatic improvement in the clozapine group. Similarly, the variability in response to treatment is the same in patients receiving clozapine relative to other antipsychotics in studies of non-TRS. CI, confidence interval; HAL, haloperidol; OLZ, olanzapine; RE, random effects; RIS, risperidone.

We, therefore, set out to address the question of whether clozapine’s superior efficacy is more evident in strictly-defined TRS, and whether this population shows a more homogenous response to clozapine relative to other antipsychotics. First, we carried out a meta-analysis of variance using data from published double-blind randomised controlled trials to test if a difference in variability of symptom change exists in TRS patients receiving clozapine compared to other antipsychotics. Next, we conducted a meta-analysis of mean difference to compare the effects of clozapine relative to other antipsychotics between patients with TRS and patients not exclusive for treatment-resistance. Finally, we used meta-regression to investigate if the rigour in definitions of TRS is a moderator of clozapine’s efficacy. Consistent with the hypothesis that clozapine shows greater efficacy for strictly-defined TRS, we hypothesised that (1) the variability in symptom change would be smaller with clozapine treatment relative to other antipsychotics in TRS patients (Fig. 1), (2) the effects of clozapine would be significantly larger in studies of TRS relative to those of patients not exclusive for TRS, and (3) clozapine would show greater efficacy in studies with more rigorous criteria for determining TRS.

## Materials and methods

This study adhered to the preferred reporting items for systematic reviews and meta-analyses (PRISMA) statement (Table S1) [18], and was registered with the PROSPERO international prospective register of systematic reviews (CRD42018086507) [19]. The full protocol, including detailed methods, is provided in the supplement. Two authors (YM and RM) independently undertook the literature search, identified eligible studies, extracted data, and assessed risk of bias of individual studies. Disagreements during these procedures were resolved through discussion with a third author (OH).

### Study selection

Studies from inception to October 18, 2018 were searched using EMBASE, Medline, PsycINFO, ClinicalTrials.gov, and Cochrane CENTRAL. Search terms included synonyms of clozapine, RCT, and schizophrenia (Supplementary Materials and Methods). No limits were applied in the search. Reference lists of relevant studies and review articles were hand-searched for additional studies. Double-blind randomised controlled trials comparing clozapine with any other antipsychotic medication in patients with schizophrenia were included. We excluded studies where the primary publication was not reported in English, single-blind or placebo-controlled trials, and studies that did not report on change in psychopathology. When there were multiple publications based on overlapping participants, the publication with the largest sample, longest duration of intervention, and/or most detailed data regarding change in psychopathology was selected.

### Data extraction

For the meta-analysis of variance, the primary outcome measure was the standard deviation (SD) of change in total symptoms as measured by the brief psychiatric rating scale (BPRS) [20] or the positive and negative syndrome scale (PANSS) [21]. For the meta-analysis of mean difference, the primary outcome measure was the mean change in total symptoms as measured by the BPRS/PANSS. Secondary outcome measures included SD of change and mean change in positive and negative symptoms as measured by the BPRS/PANSS subscales, the scale for the assessment of positive symptoms (SAPS) [22], or the scale for the assessment of negative symptoms (SANS) [23]. Furthermore, the following variables were extracted: authors, year of publication, participant characteristics (age, duration of illness, treatment setting, definition of TRS where applicable), study duration, parallel or crossover design, clozapine dose, name and dose of comparator antipsychotic, industry sponsorship, and mean ± SD total, positive, and negative symptom scores at baseline and endpoint. Doses of antipsychotics were converted into chlorpromazine equivalents (CPZE) using formulas described by Andreasen and colleagues [24]. Where relevant data were missing from the primary publication, data were extracted from related publications from the same study. Corresponding authors were contacted for additional data if SD of change in symptom scores were unreported. Only original values for SD of change in symptom scores were used in the meta-analysis of variance, as this was the primary outcome measure. For the meta-analysis of mean difference, missing values regarding SD of change were imputed (Supplementary Materials and Methods).

### Study categorisation

Studies were categorised into those strictly of TRS patients (TRS studies) and those that included patients not exclusive for TRS (non-TRS studies). To be included in the TRS group, studies were required to only include patients who were resistant to previous antipsychotic treatment. Studies which included patients with treatment-intolerance, relapse following non-adherence, or other non-treatment-resistant forms of schizophrenia were defined as non-TRS studies. TRS studies were further assessed for the rigour with which TRS was assessed by determining the number of criteria required to define TRS that were met. The TRRIP consensus minimum criteria for TRS which specify a total of eight items were used (Supplementary Materials and Methods) [7]. Criteria were weighted equally and summed to determine the total number that were used in assessment of TRS in a given study.

### Data synthesis

Risk of bias of individual studies was assessed using the Cochrane Collaboration’s Tool for Assessing Risk of Bias [25]. For the meta-analysis of variance, SD of change in symptom scores was pooled across studies to calculate the log variability ratio (lnVR) [15] using the method we applied to brain structural variability in schizophrenia [16] (Supplementary Materials and Methods).

In biological systems, the dependence between the mean and variance is common, in which larger mean values are associated with greater variance [26]. Therefore, a between-group difference in relative variability, as indexed with lnVR, may in part reflect a between-group difference in the mean. Thus, we calculated a complementary measure of relative variability which accounts for the difference in means, the log coefficient of variation ratio (lnCVR) [15]. See the supplement and Brugger and Howes 2017 [16] for a full description.

For the meta-analysis of mean difference, we calculated Hedges’ *g* to quantify between-group differences in mean effects across TRS and non-TRS studies.

### Statistical analysis

SPSS Statistics Version 24 (IBM Corp., Armonk, NY, USA) was used to compare characteristics of included studies using the Mann–Whitney *U* and chi-squared tests. Meta-analyses were performed in R 3.4.0 [27] using the metafor package [28]. Primary outcomes relating to variance and mean difference were pooled across studies using univariate random-effects models. Meta-analyses of secondary outcomes relating to positive and negative symptoms were conducted in similar fashion. To assist interpretation of findings for the meta-analysis of variance, summary effect sizes for lnVR and lnCVR were transformed back to a linear scale (Supplementary Materials and Methods) [16].

A VR (or CVR) of 1 can be interpreted as equal variability in the clozapine and other antipsychotics groups, whereas a larger (or smaller) value would indicate greater (or lower) variability in the clozapine group. For the meta-analysis of mean difference, Hedges’ *g* across TRS and non-TRS studies were compared using a Wald-type test. All statistical tests were carried out at a two-tailed alpha-level of 0.05.

### Sensitivity analysis and meta-regression

To investigate the robustness of our findings, we carried out a priori sensitivity analyses excluding studies focusing on child and adolescent patients, excluding studies with crossover designs, and for the meta-analysis of mean difference, excluding studies with imputed SD of change values. Furthermore, for the meta-analysis of mean difference, we carried out post hoc sensitivity analyses excluding studies published before 1988 when clozapine’s superiority in TRS was first demonstrated [9], disaggregating studies including both TRS and treatment-intolerant patients from non-TRS studies, categorising studies based on industry sponsorship, and examining pairwise comparisons with individual antipsychotics where two or more studies were available. For meta-regression, we tested the effects of the number of TRS criteria met, baseline symptom severity, clozapine dose, difference between CPZE dose of comparator antipsychotic and clozapine, and duration of double-blind intervention as potential moderators of effect sizes using univariate mixed-effects meta-regression. PANSS total scores were used for baseline symptom severity, with BPRS total scores converted using conversion tables [29].

### Inconsistency and publication bias

Inconsistency between studies was assessed using the *I*^*2*^ statistics, with *I*^*2*^ greater than 50% indicating moderate to high inconsistency. Publication bias was assessed by visual inspection of funnel plots and regression tests for funnel plot asymmetry.

## Results

### Study characteristics

Thirty-nine studies met inclusion criteria for the meta-analysis (Fig. S1). Characteristics of the included studies are summarised in Table 1. Ten TRS studies [9, 30–38] and 29 non-TRS studies [39–67] were included with a total of 3388 patients (822 and 2566, respectively). The average age of participants (*p* = 0.003), average dose of clozapine (*p* = 0.002), and average dose of comparator antipsychotics (*p* = 0.045) were significantly higher in TRS studies compared to non-TRS studies. When compared within study groups, the average daily dose of comparator antipsychotics in CPZE was significantly greater relative to clozapine for both TRS (*z* = 3.033, *p* = 0.002) and non-TRS (*z* = 4.099, *p* < 0.001) studies. Details regarding individual studies are summarised in Tables S2 and S3.

**Table 1:** Characteristics of included studies.

	TRS studies (*N* = 10)	Non-TRS studies (*N* = 29)	Comparisons, statistic/*p*-value
Year published, range	1988–2016	1974–2011	
Design of double-blind randomised controlled trial, *N*	Parallel 9, Crossover 1	Parallel 27, Crossover 2	
Including child and adolescent patients, *N*	0	3	
Total number of participants, median (range)	56 (13–267)	51 (15–423)	*z* = −0.290, *p* = 0.788
Average age of participants, median (range)	38.1 (35.0–42.0)	34.0 (12.3–66.5)	*z* = −2.890, *p* **=** **0.003**
Duration of double-blind intervention (weeks), median (range)	11 (6–29)	8 (4–52)	*z* = −1.170, *p* = 0.258
Total number of treatment arms compared with clozapine	12	33	
Breakdown of comparator arms (drug type and number of comparisons)	OLZ 4, HAL 3, RIS 3, CPZ 2	CPZ 9, HAL 8, OLZ 7, RIS 5, FLU 1, REM 1, ZIP 1, ZTP 1	*χ*(7) = 2.871, *p* = 0.897
Average dose of clozapine (mg/day), median (range)	490 (325–618)	304 (155–800)	*z* = −2.960, *p* **=** **0.002**
Average dose of comparator drug in CPZE^a^ (mg/day), median (range)	953 (385–1413)	503 (196–1642)	*z* = −2.007, *p* **=** **0.045**

*p*-values of <0.05 are shown in bold.

*CPZ* chlorpromazine, *CPZE* chlorpromazine equivalents, *FLU* fluphenazine, *HAL* haloperidol, *OLZ* olanzapine, *REM* remoxipride, *RIS* risperidone, *TRS* treatment-resistant schizophrenia, *ZIP* ziprasidone, *ZTP* zotepine.

^a^Calculated using formulas reported by Andreasen et al. [24]

Definitions of failed adequate treatment trials varied between TRS studies. One study [9] with rigorous criteria required inadequate response to three previous antipsychotic trials and one prospective trial with haloperidol, while two studies [34, 38] only required at least one failed antipsychotic trial. Only three TRS studies [9, 33, 35] fulfilled all the TRRIP consensus criteria [7] for adequate dose, duration, and number of previous antipsychotic trials, and none fulfilled criteria for past adherence.

Twelve studies mixed treatment-intolerant patients with treatment-resistant patients, and were, therefore, categorised as non-TRS studies [39–50]. Of the remaining 17 studies which did not select patients for TRS, patient characteristics varied with two studies including first-episode or drug-naïve patients [60, 68], while others included patients with chronic illness [57, 62, 67].

Risks for bias in individual studies are summarised in Table S4. Details regarding random sequence generation and allocation concealment were often unreported, resulting in “unclear risk” for selection bias in eight TRS (80%) and 25 non-TRS studies (86%). Similarly, few studies clearly stated blinding of outcome assessors, resulting in “unclear risk” for detection bias in eight TRS (80%) and 21 non-TRS studies (72%). Overall, only three non-TRS studies [48, 49, 62] showed “low risk” for bias in all criteria.

### Meta-analysis of variance

Fifteen studies (*n* = 1492) reporting original values for SD of change in symptom scores were included in this meta-analysis. For the primary outcome, the variability ratio (VR) for change in total symptoms did not differ significantly between clozapine and other antipsychotics in TRS [9, 30, 35, 37] (*N* = 4, VR = 1.84; 95% confidence interval (CI), 0.85–4.02; *p* = 0.124), or non-TRS studies [40, 43–48, 50, 65–67] (*N* = 11, VR = 0.98; 95%CI 0.90–1.06; *p* = 0.589) (Fig. 2a). Comparison of the pooled VR between TRS and non-TRS studies showed no significant difference (*z* = −1.587, *p* = 0.112).

There were significant positive correlations between the adjusted mean change and SD of change in total, positive and negative symptom scores (all *p* < 0.05) (Fig. S2), suggesting mean scaling of variability. However, when the coefficient of variation ratio (CVR) was used to account for the difference in means, no significant difference in variability of symptom change was observed between clozapine and other antipsychotics across TRS (CVR = 1.66; 95%CI, 0.74–3.71; *p* = 0.220), or non-TRS (CVR = 0.97; 95%CI, 0.89–1.05; *p* = 0.447) studies (Fig. 2b). There was no significant difference in CVR between TRS and non-TRS studies (*z* = 1.299, *p* = 0.194). Similarly, for the secondary outcomes of positive and negative symptoms, no significant difference in VR or CVR was found in TRS studies [9, 30, 35, 37] (Figs. S3 and S4, all *p* > 0.05). Variability in positive symptom change was significantly lower with clozapine than other antipsychotics in non-TRS studies [42–46, 48, 50, 53, 65, 66] (VR = 0.92; 95%CI, 0.84–0.99; *p* = 0.031); but not after accounting for mean-scaling (CVR = 0.90; 95%CI, 0.77–1.05; *p* = 0.170).

Sensitivity analyses relating to change in total, positive, and negative symptoms were consistent with the main results regarding VR and CVR (Table S5). The number of items fulfilled in the TRRIP consensus criteria [7] was a significant moderator of VR (*z* = 2.790, *p* = 0.005) and CVR (*z* = 2.245, *p* = 0.025), indicating that studies that met more of the TRRIP criteria for TRS were associated with greater variability in the clozapine arm for change of total symptoms (Fig. S5).

### Meta-analysis of mean difference

**Fig. 3:**
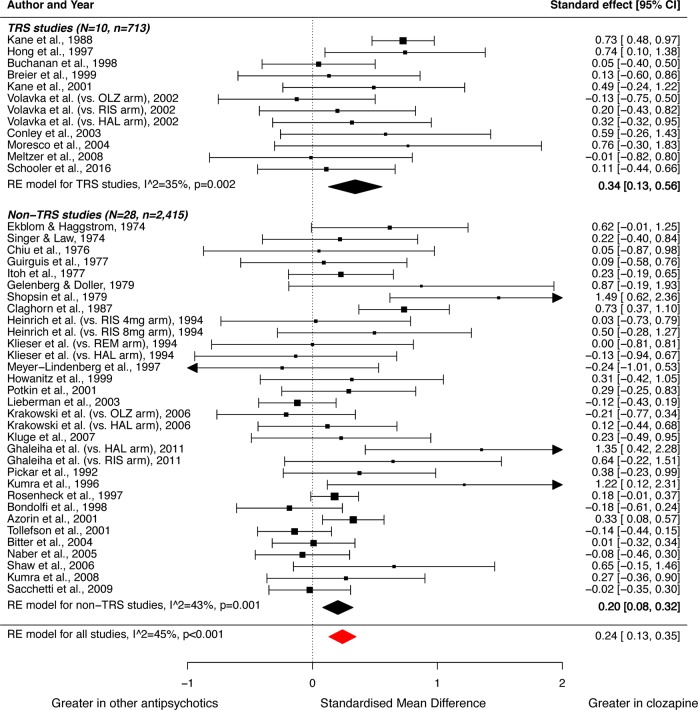
Forest plot showing the standardised mean difference (SMD) for change in total symptoms in studies of clozapine relative to other antipsychotics for the treatment of patients with strictly-defined treatment-resistant schizophrenia (TRS) and other non-refractory schizophrenia (non-TRS). Clozapine was significantly more effective in both studies of TRS (*g* = 0.34, *p* = 0.002), and non-TRS (*g* = 0.20, *p* = 0.001). CI, confidence interval; HAL, haloperidol; OLZ, olanzapine; RE, random effects; REM, remoxipride; RIS, risperidone.

For the primary outcome of total symptoms, 10 TRS studies [9, 30–38] (*n* = 713) and 28 non-TRS studies [39–60, 62–67] (*n* = 2415) were included in the meta-analysis. Clozapine was superior to other antipsychotics in improving total symptoms for both TRS (*g* = 0.34; 95%CI, 0.13–0.56; *p* = 0.002) and non-TRS (*g* = 0.20; 95%CI, 0.08–0.32; *p* = 0.001) studies (Fig. 3). For secondary outcomes, clozapine showed greater improvement in positive symptoms in both TRS (*g* = 0.32; 95%CI 0.11–0.54; *p* = 0.003) and non-TRS (*g* = 0.15; 95%CI 0.04–0.25; *p* = 0.006) studies (Fig. S6). However, this was not evident for negative symptoms in either TRS (*g* = 0.22; 95%CI −0.07–0.52; *p* = 0.135) or non-TRS (*g* = 0.07; 95%CI −0.05–0.19; *p* = 0.262) studies (Fig. S7). The comparison of the pooled effect sizes between TRS and non-TRS studies showed no significant difference for change in total symptoms (*z* = −1.105; *p* = 0.269), positive symptoms (*z* = −1.478, *p* = 0.139), or negative symptoms (*z* = −0.962; *p* = 0.336).

A priori sensitivity analyses for both TRS and non-TRS studies were largely consistent with our main finding of clozapine being superior to other antipsychotics in improving total and positive symptoms (Tables S6 and S7). However, when studies with imputed SD of change values were excluded from non-TRS studies, clozapine was no longer superior to other antipsychotics in improving total symptoms (*g* = 0.12; 95%CI −0.05–0.28; *p* = 0.178). When studies including both TRS and treatment-intolerant patients were disaggregated from the non-TRS studies, clozapine was superior in the remaining studies for improving total symptoms (*g* = 0.31; 95%CI 0.12–0.49; *p* = 0.001), but not for positive symptoms (*g* = 0.17; 95%CI −0.06–0.41; *p* = 0.154). Post hoc sensitivity analyses regarding industry sponsorship and pairwise comparisons with individual antipsychotics resulted in few studies for each comparison. The results indicate that clozapine’s superiority in improving total and positive symptoms is driven by studies sponsored by manufacturers of clozapine, and comparisons with chlorpromazine and haloperidol (Tables S6 and S7). Regarding change in total symptoms, funnel plots and regression tests indicated potential publication bias in non-TRS studies (*z* = 2.557, *p* = 0.011), but not in TRS studies (*z* = −0.734, *p* = 0.463) (Fig. S8). A trim and fill analysis suggested the presence of eight missing non-TRS studies which do not support the superiority of clozapine (Fig. S8b).

### Meta-regression

For total symptoms, mean daily dose of clozapine (*z* = 1.959, *p* = 0.050) and duration of intervention (*z* = −2.169, *p* = 0.030) were significant moderators of effect size, with greater dose of clozapine and shorter study duration associated with greater improvements with clozapine relative to other treatments (Fig. S9). The number of items fulfilled in the TRRIP consensus criteria [7] (*z* = 0.911, *p* = 0.362), baseline symptom severity (*z* = 1.679, *p* = 0.093), and difference between CPZE dose of comparator antipsychotic and clozapine (*z* = 1.733, *p* = 0.083) were not significant moderators of clozapine’s efficacy relative to other antipsychotics for total symptoms (Fig. S9). Different patterns regarding moderators of effect sizes were apparent for positive and negative symptoms (Figs. S10 and S11). TRS studies, and in particular more rigorously-defined TRS was linked to greater improvement in positive symptoms with clozapine compared to other antipsychotics (*z* = 2.168, *p* = 0.030). A higher mean dose of clozapine in trials was associated with greater improvement in both positive (*z* = 3.403, *p* < 0.001) and negative (z = 3.797, *p* < 0.001) symptoms for patients receiving clozapine. All other associations in the meta-regression were non-significant (all *p* > 0.05).

### Potential treatment-resistance in non-TRS studies

The definition of TRS requires at least two adequate trials of different antipsychotic drugs. A corollary of this is that at first presentation it is not possible to prospectively know if the illness is TRS or not, as this can only be determined retrospectively after two adequate treatment trials. Recent retrospective evidence indicates that 70–84% of patients ultimately diagnosed with TRS showed limited response from illness onset [69, 70]. This implies that non-TRS studies that included patients early in the course of illness, before it is possible to determine if the illness is treatment-resistant or not, may have included patients subsequently established to be TRS, confounding our analyses. To examine this, we carried out additional *post hoc* analyses for the non-TRS studies. First, we reviewed the non-TRS studies and excluded those which had set out to include both TRS and treatment-intolerant patients [39–50]. Of the 17 non-TRS studies remaining, two further studies were excluded due to descriptions of participants that indicated they included treatment-resistant patients [62, 63], to leave 15 studies. Of the remaining studies, fourteen reported on change in total symptoms in a total sample of 848 patients [51–60, 64–67]. We repeated the meta-analysis in this sample free of TRS patients as far as it is possible to ascertain from the trial descriptions, and found a significantly larger mean improvement in total symptoms with clozapine relative to other antipsychotics (*g* = 0.31; 95%CI 0.11–0.52; *p* = 0.003). We next examined if the duration of illness was a moderator of effect size in these studies to test if studies including more patients early in the course of illness, which may indicate a higher proportion of patients undiagnosed with TRS, showed larger effects of clozapine. However, meta-regression indicated that duration of illness in these studies was not a significant moderator of effect size (Fig. S12, *z* = −0.158, *p* = 0.874).

## Discussion

Our first main finding is that there is no significant difference in the variability in the effectiveness of clozapine compared to other antipsychotics in patients with either treatment-resistant or non-TRS, indicating similar heterogeneity in response across antipsychotics and patient groups. Our second main finding is that clozapine is superior to other antipsychotics in improving total and positive symptoms both in patients with TRS and non-TRS, with no significant difference in effect sizes between the two groups. Our third main finding is that more rigorously-defined treatment-resistance is associated with greater improvement in positive symptoms, but not total and negative symptoms, with clozapine. Our findings that variability in response is no different with clozapine, and that clozapine is more effective in non-TRS studies are contrary to our hypothesis that clozapine is specifically effective in TRS. In contrast, the finding that more rigorously-defined TRS is linked to greater improvement in positive symptoms may suggest additional benefit of clozapine in treatment-resistance.

To the best of our knowledge, there is only one meta-analysis comparing the effectiveness of clozapine relative to other antipsychotics directly between patients with treatment-resistant and non-resistant schizophrenia. Wahlbeck et al. [71] compared clozapine to first-generation antipsychotics across 30 randomised controlled trials, seven of which included treatment-resistant patients. Patients receiving clozapine were more likely to achieve “clinical improvement” as defined by individual studies, in studies of both TRS and non-resistant schizophrenia. However, it was unclear in this study how TRS studies were defined, there was no distinction between treatment-resistance and treatment-intolerance, and single-blind trials were included. While our meta-analysis supports the finding from this previous report [71], our study extends these findings by utilising international consensus guidelines on defining treatment-resistance [7], distinguishing studies including patients with treatment-intolerance, focusing on double-blind randomised controlled trials to minimise bias, and including 18 studies of second-generation antipsychotics (*n* = 1630).

Our findings are also broadly in line with a network meta-analysis by Leucht et al. [72] that showed clozapine was ranked as the most efficacious antipsychotic for acute treatment of schizophrenia in general, but not those of another network meta-analysis focusing on trials of treatment-resistance [13]. The latter study concluded that clozapine was not superior to most other drugs for TRS, particularly second-generation antipsychotics. While the conclusion regarding second-generation antipsychotics is supported by our analysis of pairwise comparisons, several methodological factors may account for the difference between findings in this study [13] and our findings. For example, the landmark study by Kane et al. [9] was excluded to resolve inconsistency in the network meta-analysis, and studies that included treatment-intolerant patients were included. Moreover, where SD of change in symptom scores were not reported, the authors extracted mean ± SD symptom scores at study endpoint while we used the reported mean change and imputed the missing SD of change value. Regarding TRS, our effect sizes for total, positive, and negative symptoms are also broadly in line with those reported by Siskind and colleagues [14] which included single-blind trials and studies including patients with treatment-intolerance.

### Strengths and limitations

The current study combined meta-analyses of variance and mean difference to investigate clozapine’s specificity in relation to TRS. Unlike previous meta-analyses, we only included double-blind randomised controlled trials to minimise bias associated with open-label treatment. Our meta-analysis was designed to address specific a priori hypotheses, and was not intended to be a comprehensive meta-analysis regarding clinical outcomes (e.g., cognitive function, relapse) and drug safety. Our meta-analysis of variance was limited by the number of studies reporting SD of change values for symptom change. Furthermore, our post hoc sensitivity analyses, particularly those regarding industry sponsorship and individual antipsychotics, resulted in a small number of studies included in each comparison. This limitation was especially relevant for TRS studies and thus findings should be interpreted in the context of limited power. Additionally, the *I*^*2*^ value indicated high inconsistency in studies included in the meta-analysis of variance. This was primarily due to a single study [30] reporting greater SD of change values in symptom change for patients treated with clozapine relative to chlorpromazine. It was unclear how the nature of this study differed from others; however, excluding this study decreased the *I*^*2*^ to <50%. Finally, we uniformly categorised studies including treatment-intolerant patients alongside treatment-resistant patients as non-TRS studies. Although it would have been ideal to extract data specifically referring to TRS patients from these studies and include them in the analysis of TRS, this was not possible from the reported data.

General limitations of the literature include the small proportion of included studies which were judged to have “low risk” of bias as double-blind trials. Furthermore, we were only able to include 10 studies of strictly-defined TRS, and only a few studies applied rigorous definitions of TRS. These limitations implicate the need for better design in future studies, with treatment-resistant patients selected according to international guidelines [7]. Another general potential issue that affects all trials and meta-analyses is that the most severely ill patients are likely to be underrepresented in double-blind randomised controlled trials. For example, only half of the studies in our meta-analysis included patients with a total PANSS equivalent of 95 or greater, which is a benchmark of “markedly ill” to “severely ill” conditions [73]. However, our meta-regression indicated that greater illness severity is not necessarily related to greater benefits with clozapine. Finally, with regard to the non-TRS studies, it is possible that studies that included patients early in the course of illness, before they had received two adequate trials of different antipsychotics, may have included patients who were subsequently established to be treatment-resistant, potentially confounding our analyses. However, we found no significant relationship between duration of illness and the effect size for clozapine over other antipsychotics, which indicates that this is unlikely to be a major confound. Moreover, when clozapine has been compared with chlorpromazine in treatment-naïve first-episode patients, before TRS can be established, there has not been a marked difference in symptom improvement between drugs [64], contrary to expectations if the inclusion of patients with unrecognised TRS in this cohort favours clozapine. Nevertheless, it would be useful if future studies of patients early in the course of their illnesses included follow-up to determine the number of patients whose illness subsequently met TRS criteria. This would provide better characterisation of samples and enable sub-analyses to test if effects of treatments are different in TRS or non-TRS groups. Another strategy could be to use biomarkers to identify patients who may have TRS to either exclude them or enrich samples for studies of TRS. There is evidence that imaging measures, such as for dopamine, glutamate and resting-state connectivity may be useful for this [74–77]. A general issue we identified in many studies is limited sample characterisation (e.g. nature of previous antipsychotic treatment and patients’ response, the proportion of patients with acute relapse following non-concordance to treatment vs. those unwell in the context of ongoing treatment). Such information is important not just to permit analyses such as ours, but also to enable clinicians to determine how generalisable study findings are to clinical practice [7]. It would be useful if these details were reported in all clinical trials of antipsychotics for these reasons.

### Interpretation and implications

Taken together, the most parsimonious explanation for our findings is that clozapine is more effective for total and positive symptoms of schizophrenia irrespective of whether patients have treatment-resistance or not. Clozapine’s unique efficacy may be attributable to a number of mechanisms including polypharmacology [78, 79] and rapid dissociation from dopamine D2 receptors [80]. While clozapine’s precise mechanism of action remains unknown, our findings suggest that this action is not necessarily specific to the neurobiology underlying treatment-resistance, and is applicable to schizophrenia in general. Of note, our findings should be interpreted in the context of potential sponsorship bias, and publication bias particularly for studies of non-resistant schizophrenia.

In terms of clinical implications, our findings show that clozapine is consistently superior in both treatment-resistant and non-resistant schizophrenia. While few moderators reached statistical significance in our meta-regression, higher clozapine doses were associated with greater benefits with clozapine for total, positive and negative symptoms. While this indicates a potential dose-response relationship, there is still limited evidence regarding the optimal dosing of clozapine [81]. Furthermore, only a few studies included in this meta-analysis [39, 43, 48, 49] reported on blood concentrations of clozapine. Observational studies show clozapine use is associated with reduced mortality in patients with schizophrenia [82], but it is generally underused in clinical care [83, 84]. A recent large-scale trial examining the effects of switching antipsychotic medications in first-episode schizophrenia found little benefit in switching between first-line antipsychotics [85]. While challenges in carrying out such studies need to be acknowledged, our findings indicate that more research is warranted on the use of clozapine in patients without established treatment-resistance, especially in early stages of illness.

## Conclusions

We did not find a systematic difference regarding variability of symptom change in TRS patients treated with clozapine and other antipsychotics. Furthermore, clozapine’s superiority in improving total and positive symptoms was not limited to patients with TRS, and was also evident in patients without established treatment-resistance. The rigour with which treatment-resistance was defined was directly associated with greater improvement in positive symptoms, but not with improvements in total or negative symptoms. Collectively, our findings do not support a subtype of schizophrenia which responds specifically to clozapine. Greater efficacy of clozapine relative to other antipsychotics was not dependent on the degree of treatment-resistance, arguing for its use more generally in schizophrenia.

## Funding and Disclosure

No funding was obtained for this study. Dr Mizuno’s research is supported by fellowship grants from Japan Society for the Promotion of Science, Astellas Foundation for Research on Metabolic Disorders, Japanese Society of Clinical Neuropsychopharmacology, and Mochida Memorial Foundation for Medical and Pharmaceutical Research. Dr Mizuno has received manuscript fees from Sumitomo Dainippon Pharma, and consultant fees from Bracket and MedAvante-ProPhase within the past three years. Dr McCutcheon’s research is funded by the Wellcome Trust (no. 200102/Z/15/Z). Dr Brugger‘s research is funded by the NIHR (no. ACF-2016-18-005). Drs McCutcheon and Brugger report no competing interests. Dr Howes has received investigator-initiated research funding from and/or participated in advisory/speaker meetings organized by Astra-Zeneca, Autifony, BMS, Eli Lilly, Heptares, Janssen, Lundbeck, Lyden-Delta, Otsuka, Servier, Sunovion, Rand and Roche. Neither Dr Howes nor his family have been employed by or have holdings/ a financial stake in any biomedical company. The views expressed here are those of the authors and do not necessarily reflect those of the NHS, the NIHR, or the Department of Health and Social Care.

## Supplementary information

Supplementary Tables 1-7

Supplementary Figures 1-4

Supplementary Figures 5-8

Supplementary Figures 9-12

Supplementary Materials and Methods
