# Supplementary material for: Heterogeneity and efficacy of antipsychotic treatment for schizophrenia with or without treatment resistance: a meta-analysis
Source: Neuropsychopharmacology. 2019 Nov 25;45(4):622–31. doi: 10.1038/s41386-019-0577-3 (PMC7021799; doi:10.1038/s41386-019-0577-3)
Supplement: Supplementary file 1 — Supplementary Tables 1-7 [file 41386_2019_577_MOESM1_ESM.doc]

**Table S1.** PRISMA 2009 checklist

| **Section/topic** | **#** | **Checklist item** | **Reported on page #** |
| --- | --- | --- | --- |
| **TITLE** | | |  |
| Title | 1 | Identify the report as a systematic review, meta-analysis, or both. | 1 |
| **ABSTRACT** | | |  |
| Structured summary | 2 | Provide a structured summary including, as applicable: background; objectives; data sources; study eligibility criteria, participants, and interventions; study appraisal and synthesis methods; results; limitations; conclusions and implications of key findings; systematic review registration number. | 3 - 4 |
| **INTRODUCTION** | | |  |
| Rationale | 3 | Describe the rationale for the review in the context of what is already known. | 5 - 7,  Supplementary Materials and Methods |
| Objectives | 4 | Provide an explicit statement of questions being addressed with reference to participants, interventions, comparisons, outcomes, and study design (PICOS). | 7 - 8, Supplementary Materials and Methods |
| **METHODS** | | |  |
| Protocol and registration | 5 | Indicate if a review protocol exists, if and where it can be accessed (e.g., Web address), and, if available, provide registration information including registration number. | 9, Supplementary Materials and Methods |
| Eligibility criteria | 6 | Specify study characteristics (e.g., PICOS, length of follow-up) and report characteristics (e.g., years considered, language, publication status) used as criteria for eligibility, giving rationale. | 9, Supplementary Materials and Methods |
| Information sources | 7 | Describe all information sources (e.g., databases with dates of coverage, contact with study authors to identify additional studies) in the search and date last searched. | 9, Supplementary Materials and Methods |
| Search | 8 | Present full electronic search strategy for at least one database, including any limits used, such that it could be repeated. | 9, Supplementary Materials and Methods |
| Study selection | 9 | State the process for selecting studies (i.e., screening, eligibility, included in systematic review, and, if applicable, included in the meta-analysis). | 9, Supplementary Materials and Methods |
| Data collection process | 10 | Describe method of data extraction from reports (e.g., piloted forms, independently, in duplicate) and any processes for obtaining and confirming data from investigators. | 9 - 10, Supplementary Materials and Methods |
| Data items | 11 | List and define all variables for which data were sought (e.g., PICOS, funding sources) and any assumptions and simplifications made. | 10, Supplementary Materials and Methods |
| Risk of bias in individual studies | 12 | Describe methods used for assessing risk of bias of individual studies (including specification of whether this was done at the study or outcome level), and how this information is to be used in any data synthesis. | 11, Supplementary Materials and Methods |
| Summary measures | 13 | State the principal summary measures (e.g., risk ratio, difference in means). | 11 - 13, Supplementary Materials and Methods |
| Synthesis of results | 14 | Describe the methods of handling data and combining results of studies, if done, including measures of consistency (e.g., I^2^) for each meta-analysis. | 12 - 14, Supplementary Materials and Methods |
| Risk of bias across studies | 15 | Specify any assessment of risk of bias that may affect the cumulative evidence (e.g., publication bias, selective reporting within studies). | 14, Supplementary Materials and Methods |
| Additional analyses | 16 | Describe methods of additional analyses (e.g., sensitivity or subgroup analyses, meta-regression), if done, indicating which were pre-specified. | 13, Supplementary Materials and Methods |
| **RESULTS** | | |  |
| Study selection | 17 | Give numbers of studies screened, assessed for eligibility, and included in the review, with reasons for exclusions at each stage, ideally with a flow diagram. | Figure S1 |
| Study characteristics | 18 | For each study, present characteristics for which data were extracted (e.g., study size, PICOS, follow-up period) and provide the citations. | 15, Tables S2 and S3 |
| Risk of bias within studies | 19 | Present data on risk of bias of each study and, if available, any outcome level assessment (see item 12). | 16, Table S4 |
| Results of individual studies | 20 | For all outcomes considered (benefits or harms), present, for each study: (a) simple summary data for each intervention group (b) effect estimates and confidence intervals, ideally with a forest plot. | Figures  2 - 3 |
| Synthesis of results | 21 | Present results of each meta-analysis done, including confidence intervals and measures of consistency. | 16 - 19 |
| Risk of bias across studies | 22 | Present results of any assessment of risk of bias across studies (see Item 15). | 19 |
| Additional analysis | 23 | Give results of additional analyses, if done (e.g., sensitivity or subgroup analyses, meta-regression [see Item 16]). | 18 - 21 |
| **DISCUSSION** | | |  |
| Summary of evidence | 24 | Summarize the main findings including the strength of evidence for each main outcome; consider their relevance to key groups (e.g., healthcare providers, users, and policy makers). | 22 |
| Limitations | 25 | Discuss limitations at study and outcome level (e.g., risk of bias), and at review-level (e.g., incomplete retrieval of identified research, reporting bias). | 24-26 |
| Conclusions | 26 | Provide a general interpretation of the results in the context of other evidence, and implications for future research. | 28 |
| **FUNDING** | | |  |
| Funding | 27 | Describe sources of funding for the systematic review and other support (e.g., supply of data); role of funders for the systematic review. | 29 |

**Table S2. Characteristics of double-blind randomised controlled trials including patients with strictly-defined treatment-resistant schizophrenia (TRS studies)**

| Author, Year^a^ | Design | Duration | Patient population | Mean (SD) age, clozapine / comparator | Mean (SD) duration of illness (years), clozapine / comparator | Mean (SD) daily dose (mg), clozapine / comparator | n, clozapine /  comparator^b^ | Type of symptom scale | Industry sponsorship |
| --- | --- | --- | --- | --- | --- | --- | --- | --- | --- |
| Kane et al., 1988 [9] | Parallel | 6 weeks | Inpatients with DSM-III SZ, at least 3 periods of treatment in the preceding 5 years with APs (from at least 2 different chemical classes) at ≥1000mg/day CPZE for 6 weeks, each without significant symptomatic relief, no period of good functioning in the preceding 5 years, non-response to prospective trial of HAL | 35.7 (8.87) | Age at first hospitalisation 20.4 (4.61) | CLO mean 618  CPZ mean 1169 | CLO 126  CPZ 141 | BPRS | Yes, supported by grants from Sandoz |
| Hong et al., 1997 [30] | Parallel | 12 weeks | Inpatients with DSM-IV SZ, persistent severe psychotic symptoms for at least 6 months while receiving adequate AP treatment (at least 2 APs from different chemical classes at ≥1000mg/day CPZE) | CLO 39.7 (8.4)  CPZ 37.1 (8.7) | Not stated | CLO 543 (157)  CPZ 1163 (228) | CLO 21  CPZ 19 | BPRS, PANSS | Not stated |
| Buchanan et al., 1998 [31] | Parallel | 10 weeks | Outpatients with DSM-III-R SZ or schizoaffective disorder, meeting retrospective criteria of residual positive and/or negative symptoms after at least two 6-week trials of therapeutic dosages of APs from different classes, minimum level of positive and/or negative symptoms at the time of evaluation for participation, and partial response to prospective trial of FLU | CLO 41.0 (6.4)  HAL 40.1 (7.9) | CLO 21.0 (5.7)  HAL 18.8 (7.0) | CLO 413.2 (59.8)  HAL 26.0 (7.0) | CLO 38  HAL 37 | BPRS, SANS | Yes, medication provided by Sandoz |
| Breier et al., 1999 [32] | Parallel | 6 weeks | Chronic DSM-IV SZ, history of residual positive and/or negative symptoms after at least a 6 week trial of a therapeutic dose of a AP, minimum level of positive and/or negative symptoms at the time of evaluation for the study, and partial response to a prospective trial of at least 2 weeks of FLU treatment | CLO 37.7 (8.6)  RIS 32.4 (11.0) | CLO 13.9 (9.5)  RIS 11.1 (9.3) | CLO 403.6 (79.6)  RIS 5.9 (1.6) | CLO 14  RIS 15 | BPRS, SANS | Not stated |
| Kane et al., 2001 [33] | Parallel | 29 weeks | Outpatients with DSM-III-R SZ or schizoaffective disorder, partial or poor response in 2 trials of conventional APs at doses of ≥600mg/day CPZE for at least 6 weeks (high-dose qualification) and 1 trial of conventional AP at 250-500mg/day CPZE for at least 6 weeks (low-dose qualification) | CLO 41 (10)  HAL 40 (8) | Years since first hospitalisation CLO 17 (11)  HAL 17 (8) | CLO 523 (171)  HAL 18.9 (7.0) | CLO 37  HAL 34 | BPRS, SANS | Yes, financial support from Novartis |
| Volavka et al., 2002 [34] | Parallel | 14 weeks | Inpatients with chronic DSM-IV SZ or schizoaffective disorder, persistent positive symptoms after at least 6 contiguous weeks of treatment with 1 or more typical APs at doses of ≥600mg/day CPZE, poor level of functioning over past 2 years, minimum PANSS score at baseline | 40.8 (9.2) | 19.5 (8.4) | CLO 526.6 (140.3)  OLZ 30.4 (6.6)  RIS 11.6 (3.2)  HAL 25.7 (5.7) | CLO 40  OLZ 39  RIS 41  HAL 37 | PANSS | Yes, Eli Lilly, Janssen, Novartis, Merck |
| Conley et al., 2003 [35] | Crossover | 8 weeks | Inpatients with DSM-IV SZ, at least 2 periods of treatment with APs from different classes at doses of ≥1000mg/day CPZE for 6 weeks without significant improvement, no period of good functioning within 5 years | CLO 40.3 (8.9)  OLZ 35.9 (9.0) | Age at first admission  CLO 18.3 (2.1)  OLZ 22.5 (6.4) | CLO 300-450  OLZ 50 | OLZ 13  CLO 10  (crossover) | BPRS | Yes, CLO supplied by Zenith-Gold-line |
| Moresco et al., 2004 [36] | Parallel | 8 weeks | Inpatients with DSM-IV SZ, lack of satisfactory clinical response to 2 APs from different classes, with duration of at least 6 weeks each and given at ≥500mg/day CPZE, minimum BPRS score required | CLO 38.3 (9.1)  OLZ 34.1 (7.6) | Not stated | CLO 325.4 (9.7)  OLZ 18.3 (0.5) | CLO 12  OLZ 11 | PANSS, BPRS | Yes, partially supported by Eli Lilly |
| Meltzer et al., 2008 [37] | Parallel | 6 months | Outpatients with DSM-IV SZ or schizoaffective disorder, moderate to severe levels of positive symptom despite 2 or more trials of typical or atypical APs from different chemical classes, with adequate doses for at least 6 weeks | CLO 37.2 (9.2)  OLZ 36.4 (11.1) | CLO 14.7 (7.8)  OLZ 16.6 (12.7) | CLO 564 (243)  OLZ 33.6 (11.2) | CLO 21  OLZ 19 | PANSS, SAPS, SANS | Yes, supported in part by grant from Eli Lilly |
| Schooler et al., 2016 [38] | Parallel | 29 weeks | Outpatients with DSM-IV SZ or schizoaffective disorder, partial or poor response defined as failure in at least 1 trial of FGA at ≥600mg/day CPZE for 6 weeks (high-dose qualification) and failure at 250-500mg/day CPZE for 4 weeks (low-dose qualification), minimum BPRS score required | CLO 42 (8)  RIS 42 (9) | Years since first hospitalization  CLO 17 (8)  RIS 18 (7) | CLO 456.7 (123.7)  RIS 6.8 (1.7) | CLO 53  RIS 54 | BPRS, SANS | Yes, Novartis and Janssen provided drugs and matching placebos |

^a^ The reference numbers correspond to those in the main text. ^b^ The number of participants randomised does not always match the number of participants included in the analysis

Abbreviations: APs, antipsychotics; BPRS, Brief Psychiatric Rating Scale; CLO, clozapine; CPZ, chlorpromazine; CPZE, chlorpromazine equivalents; DSM-III, III-R, IV, IV-TR, different versions of the Diagnostic and Statistical Manual of Mental Disorders; FGA, first-generation antipsychotic; FLU, fluphenazine; HAL, haloperidol; OLZ, olanzapine; PANSS, Positive and Negative Syndrome Scale; RIS, risperidone; SANS, Scale for the Assessment of Negative Symptoms; SAPS, Scale for the Assessment of Positive Symptoms; SD, standard deviation; SZ, schizophrenia; ZIP, ziprasidone

**Table S3.** Characteristics of double-blind randomised controlled trials including patients not exclusive for treatment-resistance (non-TRS studies)

| Author, Year^a^ | Design | Duration | Patient population | Mean (SD) age, clozapine / comparator | Mean (SD) duration of illness (years), clozapine / comparator | Mean (SD) daily dose (mg), clozapine / comparator | n, clozapine /  comparator^b^ | Type of symptom scale | Industry sponsorship |
| --- | --- | --- | --- | --- | --- | --- | --- | --- | --- |
| ***Studies mixing treatment-intolerant patients with treatment-resistant patients*** | | | | | | | | | |
| Pickar et al., 1992 [39] | Crossover | CLO 51.8 (14.7) days  FLU 45.8 (15.8) days | Inpatients with DSM-III-R SZ or schizoaffective illness, history of drug intolerance defined as significant tardive dyskinesia or EPS, or being refractory to at least 2 different APs given at adequate doses and periods | 29.4 (6.1) | 11.3 (6.4) | CLO 373.8 (110.8)  FLU 28.9 (21.2) | 21 (crossover) | BPRS | Yes , supported by Sandoz |
| Kumra et al., 1996 [40] | Parallel | 6 weeks | Inpatients with childhood-onset DSM-III-R SZ, 6-18 years of age, history of intolerance, non-response, or both to at least 2 different APs | CLO 14.4 (3.0)  HAL 13.7 (1.6) | CLO 4.8 (1.9)  HAL 3.4 (1.6) | CLO 176 (149)  HAL 16 (8) | CLO 10  HAL 11 | BPRS, SAPS, SANS | Yes, blinded medication assisted by Sandoz |
| Meyer-  Lindenberg et al., 1997 [41] | Parallel | 6 weeks | Patients with DSM-III-R SZ, treated before for at least 3 weeks each with 2 conventional APs in effective doses without satisfactory results or with intolerable side-effects | CLO 32.6 (9.5)  ZTP 33.7 (10.0) | Age at onset CLO 26.0 (6.2)  ZTP 27.8 (9.0) | CLO max 450  ZTP max 450 | CLO 25  ZTP 25 | BPRS, SANS | Yes, sponsored by Klinge Pharma |
| Rosenheck et al., 1997 [42] | Parallel | 12 months | Inpatients with DSM-III-R SZ, persistent psychotic symptoms despite receiving adequate treatment trials of 2 or more APs at 1000mg/day CPZE unless limited by adverse effects, severe symptoms indicated by BPRS and CGI, and serious social dysfunction for previous 2 years | CLO 43.2 (7.7)  HAL 43.9 (8.3) | Age at onset  CLO 22.2 (5.8)  HAL 22.4 (4.9) | CLO 552 (229)  HAL 28 (5.3) | CLO 205  HAL 218 | PANSS | Yes, CLO provided by Sandoz |
| Bondolfi et al., 1998 [43] | Parallel | 8 weeks | Inpatients with chronic DSM-III-R SZ, failure to respond to or intolerant of at least 2 different classes of APs given in appropriate doses for at least 4 weeks each, total score of 60-120 required on PANSS | CLO 36.2 (12.2)  RIS 38.3 (12.9) | Age at onset CLO 23.5 (7.4)  RIS 22.4 (6.8) | CLO mean 291.2  RIS mean 6.4 | CLO 43  RIS 43 | PANSS | Yes, grant from Janssen; medication supplied by Janssen and Novartis |
| Azorin et al., 2001 [44] | Parallel | 12 weeks | Inpatients and outpatients with DSM-IV SZ, minimum CGI and BPRS upon entry, no significant improvement with continuous treatment with AP for at least the preceding 6 months, unsuccessful trial of AP equivalent to 20mg/day HAL for at least 6 weeks in current episode (less if the patient had dose-limiting adverse events), no period of good functioning for at least 24 months | CLO 37.8 (10.4)  RIS 39.5 (11.3) | CLO median 13.0  RIS median 15.5 | CLO median 600  RIS median 6 | CLO 138  RIS 135 | PANSS, BPRS | Yes, supported by grants from Novartis |
| Tollefson et al., 2001 [45] | Parallel | 18 weeks | Inpatients or outpatients with DSM-IV SZ, minimum defined score on BPRS, lack of satisfactory response to at least 2 oral APs from different chemical classes, given for at least 6 weeks at doses of ≥500mg/day CPZE or to maximum daily dose in case of intolerable side effects | 38.6 (10.6) | Age of onset 22.8 (5.6) | CLO 303.6 (108.7)  OLZ 20.5 (2.8) | CLO 90  OLZ 90 | PANSS, BPRS | Yes, reported by employees of Eli Lilly |
| Bitter et al., 2004 [46] | Parallel | 18 weeks | Inpatients with DSM-IV SZ, minimum BPRS score required at screening, insufficient effectiveness or intolerable side effects to at least 1 treatment trial with conventional APs of 4-6 weeks duration at 400-600mg/day CPZE | Mean 37.6 | Not stated | CLO 216.2 (107.9)  OLZ 17.2 (4.8) | CLO 72  OLZ 75 | PANSS | Yes, sponsored by Eli Lilly |
| Naber et al., 2005 [47] | Parallel | 26 weeks | Patients with DSM-IV SZ, history of failure to respond to at least 1 AP or had experience of intolerable side effects during these prior AP treatments | CLO 35.2 (10.8)  OLZ 32.9 (10.4) | Age at onset  CLO 26.8 (6.5)  OLZ 27.0 (8.9) | CLO 209 (91)  OLZ 16.2 (4.8) | CLO 57  OLZ 57 | PANSS, BPRS | Yes, funded by Eli Lilly |
| Shaw et al., 2006 [48] | Parallel | 8 weeks | Children aged 7-16 years, inpatients with DSM-IV SZ, failure to respond to at least 2 APs at greater than 100mg/day CPZE and ≥4 weeks duration unless terminated owing to intolerable adverse effects | CLO 11.7 (2.3)  OLZ 12.8 (2.4) | CLO 3.1 (1.9)  OLZ 3.3 (3.0) | CLO 327 (113)  OLZ 18.1 (4.3) | CLO 12  OLZ 13 | BPRS, SAPS, SANS | Not stated |
| Kumra et al., 2008 [49] | Parallel | 12 weeks | Children aged 10-18 years, predominantly inpatients, diagnosed with DSM-IV SZ or schizoaffective disorder, treatment-refractory or intolerant to at least 2 adequate trials of APs, minimum BPRS score at baseline | CLO 15.8 (2.2)  OLZ 15.5 (2.1) | CLO mean 3.1  OLZ mean 3.8 | CLO 403.1 (201.8)  OLZ 26.2 (6.5) | CLO 18  OLZ 21 | BPRS, SANS | None |
| Sacchetti et al., 2009 [50] | Parallel | 18 weeks | Patients with DSM-IV SZ, resistant and/or intolerant to at least 3 acute cycles with different APs in previous 5 years, each with therapeutic doses proposed by manufacturer and at least 6-week duration unless intolerant | CLO 38.3 (11.2)  ZIP 41.6 (10.2) | CLO mean 14.1  ZIP mean 13.4 | CLO 346 (61)  ZIP 130 (24) | CLO 74  ZIP 73 | PANSS | Yes, Pfizer provided funding, support and staff |
| ***Studies including patients with other non-refractory schizophrenia*** | | | | | | | | | |
| Ekblom & Haggstrom, 1974 [51] | Parallel | 40 days | Hospitalised male patients with acute SZ, and relapsed or exacerbated chronic SZ (diagnostic criteria not stated) | CLO mean 33  CPZ mean 28 | CLO mean 6.2  CPZ mean 3.5 | CLZ range 279-338  CPZ range 320-410 | CLO 20  CPZ 21 | BPRS | Not stated |
| Singer & Law, 1974 [52] | Parallel | 40 days | Inpatients with SZ with acute symptomatology (diagnostic criteria not stated) | CLO mean 32.1  CPZ mean 31.6 | CLO mean 1.7  CPZ mean 3.3 | CLO mean 155  CPZ mean 196 | CLO 20  CPZ 20 | BPRS | Not stated |
| Chiu et al., 1976 [53] | Parallel | 6 weeks | Inpatients with acute episodes of schizophrenic illness of moderate to severe symptomatology (diagnostic criteria not stated) | CLO mean 32.6  CPZ mean 30 | Mean 6.4 weeks | CLO 300  CPZ 300 | CLO 33  CPZ 31 | BPRS | Yes, material provided by Sandoz |
| Guirguis et al., 1977 [54] | Parallel | 7 weeks | Inpatients with SZ (diagnostic criteria not stated), either previously untreated acute SZ, acutely relapsed SZ, or acute exacerbations in chronic SZ | CLO 41.5 (2.5)  CPZ 34.7 (2.0) | Age at first signs of SZ  CLO 33.4 (2.5)  CPZ 26.3 (1.5) | CLO range 75-450  CPZ range 150-900 | CLO 22  CPZ 28 | BPRS | Yes, sponsored by Sandoz |
| Itoh et al., 1977 [55] | Parallel | 12 weeks | Inpatients with SZ (diagnostic criteria not stated) | Not stated | Not stated | CLO max 500  HAL max 15 | CLO 47  HAL 41 | BPRS | Not stated |
| Gelenberg & Doller, 1979 [56] | Parallel | 4 weeks | Inpatients with an acute psychotic episode of DSM-II SZ, minimum criteria of BPRS scores, history of neurologic reaction associated with previous AP use | CLO mean 28.3  CPZ mean 30.8 | Not stated | CLO mean 279  CPZ mean 606 | CLO 7  CPZ 8 | BPRS | Yes, funded in part by Sandoz |
| Shopsin et al., 1979 [57] | Parallel | 5 weeks | Inpatients with acute schizophrenic symptoms (diagnostic criteria not stated), largely a chronically ill population with recent history of acute symptom exacerbation, minimum criteria for symptom profile | Not stated | Not stated | CLO range 300- 800  CPZ range 600-1800 | CLO 13  CPZ 12 | BPRS | Not stated |
| Claghorn et al., 1987 [58] | Parallel | 8 weeks | Inpatients with DSM-II SZ, exhibiting neurological reactions (either tardive dyskinesia or EPS) induced by prior medication with at least 2 different APs | Median 30 | Not stated | CLO mean 390  CPZ mean 698 | CLO 75  CPZ 76 | BPRS | Not stated |
| Heinrich et al., 1994 [59] | Parallel | 4 weeks | Acute patients with SZ or schizoaffective psychosis (diagnostic criteria not stated) | CLO median 38.0  RIS 4mg median 32.5  RIS 8mg median 29.0 | Not stated | CLO 400  RIS 4  RIS 8 | CLO 20  RIS 4mg 20  RIS 8mg 19 | BPRS | Not stated |
| Klieser et al., 1994 [60] | Parallel | 4 weeks | Patients with acute ICD-9 SZ who had not received any AP pretreatment | CLO 31.1 (11.1)  Remoxipride 32.6 (10.8)  HAL 33.7 (9.7) | CLO 4.9 (5.2)  Remoxipride 6.0 (5.6)  HAL 5.1 (4.6) | CLO 350 (75)  Remoxipride 375 (100)  HAL 16 (8) | CLO 17  Remoxipride 17  HAL 17 | BPRS | Not stated |
| Tamminga et al., 1994 [61] | Parallel | 12 months | Patients with SZ (diagnostic criteria not stated) diagnosed with tardive dyskinesia of a moderate to severe degree | CLO 35.4 (31.6)  HAL 35.8 (28.7) | CLO 10.6 (25.6) months  HAL 11.6 (25.6) months | CLO 293.8 (171.9)  HAL 28.5 (23.8) | CLO 19  HAL 13 | BPRS | Not stated |
| Howanitz et al., 1999 [62] | Parallel | 12 weeks | Geriatric inpatients, aged 55 or greater, with chronic DSM-IV SZ or schizoaffective disorder, minimum criteria for total PANSS score | CLO mean 65  CPZ mean 68.5 | CLO mean 38  CPZ mean 40 | CLO 300  CPZ 600 | CLO 24  CPZ 18 | PANSS | Not stated |
| Potkin et al., 2001 [63] | Crossover | 5-6 weeks | Inpatients with DSM-III-R SZ, two thirds of the patients were refractory to standard APs (no definition given) | Mean 34.9 | Mean 15.9 | CLO 300-500  HAL adjusted to plasma concentration of 10-20μg/mL | 27 (crossover) | BPRS, SANS | Not stated |
| Lieberman et al., 2003 [64] | Parallel | 52 weeks | Inpatients with first-episode DSM-IV SZ or schizophreniform disorder, duration of symptoms no longer than 60 months, no prior treatment with APs (or total lifetime usage of less than 14 days), minimum criteria for BPRS score | 28.7 (6.9) | 18.4 (17.8) months | CLO median 300  CPZ median 400 | CLO 81  CPZ 83 | BPRS, SANS | Yes, supported by Novartis |
| Krakowski et al., 2006 [65] | Parallel | 12 weeks | Inpatients with DSM-IV SZ or schizoaffective disorder, with a clearly confirmed episode of physical assault directed at another person during current hospitalisation and some persistence of aggression, as evidenced by the presence of some other aggressive event, whether physical or verbal or against property | CLO 35.1 (12.3)  OLZ 35.6 (9.4)  HAL 32.7 (10.6) | CLO 15.7 (9.5)  OLZ 16.8 (11.2)  HAL 13.9 (11.2) | CLO 565.5 (112.7)  OLZ 24.7 (6.1)  HAL 23.3 (7.1) | CLO 37  OLZ 37  HAL 36 | PANSS | Yes, Eli Lilly & Novartis provided medications, Eli Lilly provided supplemental funding |
| Kluge et al., 2007 [66] | Parallel | 6 weeks | Inpatients with DSM-IV SZ, schizophreniform, or schizoaffective disorder, minimum criteria for BPRS score | CLO 36.7 (13.0)  OLZ 32.8 (8.3) | Age at onset  CLO 30 (8)  OLZ 28 (11) | CLO 266.7 (77.9)  OLZ 21.2 (2.5) | CLO 15  OLZ 15 | BPRS | Yes, funded by Eli Lilly |
| Ghaleiha et al., 2011 [67] | Parallel | 8 weeks | Inpatients with chronic DSM-IV-TR SZ in the active phase of illness, minimum criteria for PANSS total score | HAL 33.0 (10.1)  CLO 37.1 (9.4)  RIS 32.1 (5.8) | HAL 7.3 (4.3)  CLO 7.8 (5.9)  RIS 8.0 (6.0) | HAL 15  CLO 300  RIS 6 | HAL 17  CLO 17  RIS 17 | PANSS | None |

^a^ The reference numbers correspond to those in the main text. ^b^ The number of participants randomised does not always match the number of participants included in the analysis

Abbreviations: APs, antipsychotics; BPRS, Brief Psychiatric Rating Scale; CLO, clozapine; CPZ, chlorpromazine; CPZE, chlorpromazine equivalents; DSM-II, III, III-R, IV, IV-TR, different versions of the Diagnostic and Statistical Manual of Mental Disorders; EPS, extrapyramidal symptoms; HAL, haloperidol; ICD, International Classification of Diseases; OLZ, olanzapine; PANSS, Positive and Negative Syndrome Scale; RIS, risperidone; SANS, Scale for the Assessment of Negative Symptoms; SAPS, Scale for the Assessment of Positive Symptoms; SD, standard deviation; SZ, schizophrenia; ZTP, zotepine

**Table S4. Risk of bias summary of individual studies**

| **Author, Year**^a^ | **Selection bias:** Random sequence generation | **Selection bias:** Allocation concealment | **Performance bias:** Blinding of participants and personnel | **Detection bias:** Blinding of outcome assessment | **Attrition bias:** Incomplete outcome data addressed | **Reporting bias:** Selective reporting | **Other bias:** ^b^ |
| --- | --- | --- | --- | --- | --- | --- | --- |
| ***TRS studies*** |  |  |  |  |  |  |  |
| Kane et al., 1988 [9] | Unclear | Unclear | Low | Low | Low | Low | Low |
| Hong et al., 1997 [30] | Low | Unclear | Low | Unclear | Low | Low | Low |
| Buchanan et al., 1998 [31] | Low | Unclear | Low | Unclear | Low | Low | Low |
| Breier et al., 1999 [32] | Unclear | Unclear | Low | Unclear | Low | Low | Low |
| Kane et al., 2001 [33] | Low | Low | Low | Unclear | Low | Low | Low |
| Volavka et al., 2002 [34] | Unclear | Unclear | Low | Low | Low | Low | Low |
| Conley et al., 2003 [35] | Unclear | Unclear | Low | Unclear | Low | Low | Low |
| Moresco et al., 2004 [36] | Unclear | Unclear | Low | Unclear | Low | Low | Low |
| Meltzer et al., 2008 [37] | Low | Low | Low | Unclear | Low | Low | Low |
| Schooler et al., 2016 [38] | Unclear | Unclear | Low | Unclear | Unclear | Low | Low |
| ***Non-TRS studies*** |  |  |  |  |  |  |  |
| *Studies mixing treatment-intolerant patients with treatment-resistant patients* | | | | | | | |
| Pickar et al., 1992 [39] | Unclear | Unclear | Low | Low | Low | Low | Low |
| Kumra et al., 1996 [40] | Low | Low | Low | Unclear | Low | Low | Low |
| Meyer-Lindenberg et al., 1997 [41] | Unclear | Unclear | Low | Unclear | Low | Low | Low |
| Rosenheck et al., 1997 [42] | Unclear | Unclear | Low | Unclear | Low | Low | Low |
| Bondolfi et al., 1998 [43] | Unclear | Unclear | Low | Unclear | Low | Low | Low |
| Azorin et al., 2001 [44] | Low | Unclear | Low | Unclear | Low | Low | Low |
| Tollefson et al., 2001 [45] | Unclear | Unclear | Low | Unclear | Low | Low | Low |
| Bitter et al., 2004 [46] | Low | Unclear | Low | Unclear | Low | Low | Low |
| Naber et al., 2005 [47] | Unclear | Unclear | Low | Unclear | Low | Low | Low |
| Shaw et al., 2006 [48] | Low | Low | Low | Low | Low | Low | Low |
| Kumra et al., 2008 [49] | Low | Low | Low | Low | Low | Low | Low |
| Sacchetti et al., 2009 [50] | Unclear | Low | Low | Unclear | Low | Low | Low |
| *Studies including patients with other non-refractory schizophrenia* | | | | | | | |
| Ekblom & Haggstrom, 1974 [51] | Unclear | Unclear | Low | Unclear | Low | Low | Unclear |
| Singer & Law, 1974 [52] | Unclear | Unclear | Low | Unclear | Low | Low | Unclear |
| Chiu et al., 1976 [53] | Unclear | Unclear | Low | Unclear | Unclear | Low | Unclear |
| Guirguis et al., 1977 [54] | Unclear | Low | Low | Unclear | Low | Low | Unclear |
| Itoh et al., 1977 [55] | Unclear | Low | Low | Unclear | Unclear | Low | Unclear |
| Gelenberg & Doller, 1979 [56] | Unclear | Unclear | Low | Unclear | Unclear | Low | Unclear |
| Shopsin et al., 1979 [57] | Unclear | Unclear | Low | Low | Low | Low | Unclear |
| Claghorn et al., 1987 [58] | Unclear | Unclear | Low | Unclear | Low | Low | Low |
| Heinrich et al., 1994 [59] | Unclear | Unclear | Low | Unclear | Low | Low | Unclear |
| Klieser et al., 1994 [60] | Unclear | Unclear | Low | Unclear | Low | Low | Low |
| Tamminga et al., 1994 [61] | Unclear | Low | Low | Low | Unclear | Low | Unclear |
| Howanitz et al., 1999 [62] | Low | Low | Low | Low | Low | Low | Low |
| Potkin et al., 2001 [63] | Unclear | Unclear | Low | Low | Low | Low | Low |
| Lieberman et al., 2003 [64] | Unclear | Unclear | Low | Unclear | Low | Low | Low |
| Krakowski et al., 2006 [65] | Low | Unclear | Low | Low | Low | Low | Low |
| Kluge et al., 2007 [66] | Unclear | Unclear | Low | Unclear | Low | Low | Low |
| Ghaleiha et al., 2011 [67] | Low | Unclear | Low | Unclear | Low | Low | Low |

Assessed using the Cochrane Collaboration’s Tool for Assessing Risk of Bias, with blue indicating “low risk”, red indicating “high risk”, and white indicating “unclear risk” of bias.

^a^ The reference numbers correspond to those in the main text. ^b^ Based on use of widely accepted diagnostic criteria (i.e. Diagnostic and Statistical Manual of Mental Disorders, DSM; International Statistical Classification of Diseases and Related Health Problems, ICD)

**Table S5.** Sensitivity analysis regarding VR/CVR of change in total, positive, and negative symptoms

| Analysis | **VR** | 95% CI | *I^2^* | *p*-value | **CVR** | 95% CI | *I^2^* | *p*-value |
| --- | --- | --- | --- | --- | --- | --- | --- | --- |
| ***Total symptoms*** |  |  |  |  |  |  |  |  |
| #1 Original analysis, TRS studies (N=4, n=352) | 1.84 | 0.85 – 4.02 | 93% | 0.124 | 1.66 | 0.74 – 3.71 | 94% | 0.220 |
| #1.1 Excluding child/adolescent patients | NA | | | | NA | | | |
| #1.2 Excluding studies with crossover design (N=3, n=329) | 2.07 | 0.73 – 5.84 | 95% | 0.170 | 1.89 | 0.65 – 5.47 | 96% | 0.241 |
| #2 Original analysis, non-TRS studies (N=11, n=1,140) | 0.98 | 0.90 – 1.06 | 0% | 0.589 | 0.97 | 0.89 – 1.05 | 2% | 0.447 |
| #2.1 Excluding child/adolescent patients (N=9, n=1,099) | 0.98 | 0.90 – 1.07 | 0% | 0.699 | 0.98 | 0.90 – 1.07 | 1% | 0.495 |
| #2.2 Excluding studies with crossover design | NA | | | | NA | | | |
| ***Positive symptoms*** |  | | | |  | | | |
| #3 Original analysis, TRS studies (N=4, n=352) | 1.61 | 0.80 – 3.27 | 91% | 0.185 | 1.21 | 0.82 – 1.80 | 84% | 0.334 |
| #3.1 Excluding child/adolescent patients | NA | | | | NA | | | |
| #3.2 Excluding studies with crossover design (N=3, n=329) | 1.80 | 0.71 – 4.57 | 94% | 0.216 | 1.27 | 0.76 – 2.12 | 90% | 0.371 |
| #4 Original analysis, non-TRS studies (N=10, n=1,221) | 0.92 | 0.84 – 0.99 | 0% | **0.031** | 0.90 | 0.77 – 1.05 | 83% | 0.170 |
| #4.1 Excluding child/adolescent patients (N=9, n=1,196) | 0.92 | 0.85 – 1.00 | 1% | 0.052 | 0.94 | 0.84 – 1.05 | 68% | 0.268 |
| #4.2 Excluding studies with crossover design | NA | | | | NA | | | |
| ***Negative symptoms*** |  | | | |  | | | |
| #5 Original analysis, TRS studies (N=4, n=351) | 1.61 | 0.88 – 2.95 | 88% | 0.122 | 1.88 | 0.94 – 3.77 | 91% | 0.074 |
| #5.1 Excluding child/adolescent patients | NA | | | | NA | | | |
| #5.2 Excluding studies with crossover design (N=3, n=328) | 1.81 | 0.84 – 3.91 | 91% | 0.129 | NA | | | |
| #6 Original analysis, non-TRS studies (N=11, n=1,311) | 1.02 | 0.94 – 1.10 | 0% | 0.620 | 1.00 | 0.93 – 1.08 | 34% | 0.950 |
| #6.1 Excluding child/adolescent patients (N=9, n=1,286) | 1.02 | 0.94 – 1.10 | 1% | 0.648 | 1.01 | 0.93 – 1.10 | 50% | 0.725 |
| #6.2 Excluding studies with crossover design | NA | | | | NA | | | |

VR and CVR values represent ratios, with values larger (or smaller) than 1 representing greater (or less) variability in change of total symptoms in patients receiving clozapine relative to other antipsychotics. Abbreviations: CI, confidence interval; CVR, coefficient of variation ratio; NA, not applicable; TRS, treatment resistant schizophrenia; VR, variability ratio.

**Table S6.** Sensitivity analysis regarding SMD for change in total, positive, and negative symptoms in TRS studies

| Analysis | **SMD** | 95% CI | *I^2^* | *p*-value |
| --- | --- | --- | --- | --- |
| ***Total symptoms*** |  |  |  |  |
| #1 Original analysis, TRS studies (N=10, n=713) | 0.34 | 0.13 – 0.56 | 35% | **0.002** |
| #1.1 Excluding child/adolescent patients | NA | | | |
| #1.2 Excluding studies with crossover design (N=9, n=690) | 0.33 | 0.10 – 0.55 | 39% | **0.004** |
| #1.3 Excluding studies with imputed SD of change values (N=4, n=352) | 0.67 | 0.45 – 0.88 | 0% | **<0.001** |
| # 1.4 Excluding studies published before 1988 | NA | | | |
| # 1.5 Categorisation based on industry sponsorship |  |  |  |  |
| No industry sponsorship or sponsorship status unreported (N=2, n=69) | 0.46 | -0.13 – 1.06 | 34% | 0.128 |
| Sponsored by manufacturer of clozapine (N=4, n=397) | 0.47 | 0.10 – 0.84 | 53% | **0.013** |
| Sponsored by manufacturer of comparator antipsychotic (N=2, n=39) | 0.30 | -0.45 – 1.04 | 22% | 0.438 |
| Sponsored by both manufacturers of clozapine and comparator antipsychotic (N=2, n=208) | 0.12 | -0.18 – 0.43 | 0% | 0.425 |
| # 1.6 Comparison with individual antipsychotics |  |  |  |  |
| vs. chlorpromazine (N=2, n=305) | 0.73 | 0.50 – 0.96 | 0% | **<0.001** |
| vs. haloperidol (N=3, n=159) | 0.21 | -0.12 – 0.54 | 0% | 0.209 |
| vs. olanzapine (N=4, n=114) | 0.19 | -0.22 – 0.61 | 7% | 0.367 |
| vs. risperidone (N=3, n=135) | 0.15 | -0.21 – 0.50 | 0% | 0.428 |
| ***Positive symptoms*** |  |  |  |  |
| #2 Original analysis, TRS studies (N=10, n=713) | 0.32 | 0.11 – 0.54 | 34% | **0.003** |
| #2.1 Excluding child/adolescent patients | NA | | | |
| #2.2 Excluding studies with crossover design (N=9, n=690) | 0.32 | 0.10 – 0.54 | 37% | **0.005** |
| #2.3 Excluding studies with imputed SD of change values (N=4, n=352) | 0.68 | 0.46 – 0.89 | 0% | **<0.001** |
| # 2.4 Excluding studies published before 1988 | NA | | | |
| # 2.5 Categorisation based on industry sponsorship |  |  |  |  |
| No industry sponsorship or sponsorship status unreported (N=2, n=69) | 0.57 | -0.04 – 1.18 | 36% | 0.066 |
| Sponsored by manufacturer of clozapine (N=4, n=397) | 0.42 | 0.07 – 0.76 | 47% | **0.017** |
| Sponsored by manufacturer of comparator antipsychotic (N=2, n=39) | 0.59 | -0.06 – 1.24 | 0% | 0.077 |
| Sponsored by both manufacturers of clozapine and comparator antipsychotic (N=2, n=208) | 0.02 | -0.28 – 0.32 | 0% | 0.905 |
| # 2.6 Comparison with individual antipsychotics |  |  |  |  |
| vs. chlorpromazine (N=2, n=305) | 0.70 | 0.47 – 0.93 | 0% | **<0.001** |
| vs. haloperidol (N=3, n=159) | 0.13 | -0.20 – 0.46 | 0% | 0.430 |
| vs. olanzapine (N=4, n=114) | 0.27 | -0.13 – 0.67 | 0% | 0.179 |
| vs. risperidone (N=3, n=135) | 0.09 | -0.27 – 0.45 | 0% | 0.631 |
| ***Negative symptoms*** |  |  |  |  |
| #3 Original analysis, TRS studies (N=6, n=528) | 0.22 | -0.07 – 0.52 | 47% | 0.135 |
| #3.1 Excluding child/adolescent patients | NA | | | |
| #3.2 Excluding studies with crossover design | NA | | | |
| #3.3 Excluding studies with imputed SD of change values (N=3, n=328) | 0.38 | -0.18 – 0.94 | 68% | 0.189 |
| # 3.4 Excluding studies published before 1988 | NA | | | |
| # 3.5 Categorisation based on industry sponsorship |  |  |  |  |
| No industry sponsorship or sponsorship status unreported (N=2, n=69) | 0.21 | -0.54 – 0.96 | 59% | 0.577 |
| Sponsored by manufacturer of clozapine (N=1, n=264) | NA | | | |
| Sponsored by manufacturer of comparator antipsychotic (N=2, n=39) | -0.33 | -0.97 – 0.31 | 0% | 0.314 |
| Sponsored by both manufacturers of clozapine and comparator antipsychotic (N=1, n=156) | NA | | | |
| # 3.6 Comparison with individual antipsychotics |  |  |  |  |
| vs. chlorpromazine (N=2, n=304) | 0.64 | 0.41 – 0.87 | 0% | **<0.001** |
| vs. haloperidol (N=1, n=50) | NA | | | |
| vs. olanzapine (N=3, n=91) | -0.16 | -0.61 – 0.29 | 0% | 0.481 |
| vs. risperidone (N=2, n=83) | 0.02 | -0.45 – 0.50 | 0% | 0.924 |

SMD values are shown as effect sizes, with values larger (or smaller) than 0 representing greater (or less) improvement of symptoms in patients receiving clozapine compared to other antipsychotics. *P*-values of <0.05 are shown in bold. Abbreviations: CI, confidence interval; NA, not applicable; SD, standard deviation; SMD, standardised mean difference; TRS, treatment-resistant schizophrenia.

**Table S7.** Sensitivity analysis regarding SMD for change in total, positive, and negative symptoms in non-TRS studies

| Analysis | **SMD** | 95% CI | *I^2^* | *p*-value |
| --- | --- | --- | --- | --- |
| ***Total symptoms*** |  |  |  |  |
| #1 Original analysis, non-TRS studies (N=28, n=2,415) | 0.20 | 0.08 – 0.32 | 43% | **0.001** |
| #1.1 Excluding child/adolescent patients (N=25, n=2,335) | 0.18 | 0.06 – 0.30 | 44% | **0.004** |
| #1.2 Excluding studies with crossover design (N=26, n=2,319) | 0.20 | 0.07 – 0.33 | 47% | **0.003** |
| #1.3 Excluding studies with imputed SD of change values (N=11, n=1,140) | 0.12 | -0.05 – 0.28 | 40% | 0.178 |
| #1.4 Excluding studies published before 1988 (N=20, n=2,027) | 0.11 | 0.00 – 0.21 | 19% | **0.048** |
| # 1.5 Disaggregating studies including both TRS and treatment-intolerant patients from non-TRS studies |  |  |  |  |
| Studies including both TRS and treatment-intolerant patients (N=12, n=1,481) | 0.10 | -0.04 – 0.23 | 28% | 0.153 |
| Other non-TRS studies (N=16, n=934) | 0.31 | 0.12 – 0.49 | 41% | **0.001** |
| # 1.6 Categorisation based on industry sponsorship |  |  |  |  |
| No industry sponsorship or sponsorship status unreported (N=12, n=629) | 0.45 | 0.27 – 0.64 | 18% | **<0.001** |
| Sponsored by manufacturer of clozapine (N=8, n=965) | 0.21 | 0.03 – 0.39 | 29% | **0.025** |
| Sponsored by manufacturer of comparator antipsychotic (N=6, n=624) | -0.06 | -0.21 – 0.10 | 0% | 0.480 |
| Sponsored by both manufacturers of clozapine and comparator antipsychotic (N=2, n=197) | -0.11 | -0.40 – 0.18 | 0% | 0.454 |
| # 1.7 Comparison with individual antipsychotics |  |  |  |  |
| vs. chlorpromazine (N=9, n=492) | 0.42 | 0.10 – 0.74 | 60% | **0.010** |
| vs. haloperidol (N=7, n=686) | 0.23 | 0.08 – 0.38 | 0% | **0.003** |
| vs. olanzapine (N=7, n=574) | -0.02 | -0.18 – 0.15 | 0% | 0.824 |
| vs. risperidone (N=4, n=425) | 0.21 | -0.09 – 0.50 | 36% | 0.166 |
| ***Positive symptoms*** |  |  |  |  |
| #2 Original analysis, non-TRS studies (N=15, n=1,436) | 0.15 | 0.04 – 0.25 | 0% | **0.006** |
| #2.1 Excluding child/adolescent patients (N=13, n=1,372) | 0.13 | 0.03 – 0.24 | 0% | **0.015** |
| #2.2 Excluding studies with crossover design (N=13, n=1,340) | 0.15 | 0.04 – 0.25 | 0% | **0.008** |
| #2.3 Excluding studies with imputed SD of change values (N=10, n=1,221) | 0.14 | 0.02 – 0.26 | 6% | **0.020** |
| #2.4 Excluding studies published before 1988 (N=14, n=1,418) | 0.15 | 0.04 – 0.25 | 0% | **0.007** |
| # 2.5 Disaggregating studies including both TRS and treatment-intolerant patients from non-TRS studies |  |  |  |  |
| Studies including both TRS and treatment-intolerant patients (N=9, n=1,143) | 0.14 | 0.01 – 0.26 | 9% | **0.032** |
| Other non-TRS studies (N=6, n=293) | 0.17 | -0.06 – 0.41 | 0% | 0.154 |
| # 2.6 Categorisation based on industry sponsorship |  |  |  |  |
| No industry sponsorship or sponsorship status unreported (N=5, n=198) | 0.24 | -0.05 – 0.53 | 0% | 0.100 |
| Sponsored by manufacturer of clozapine (N=4, n=551) | 0.25 | 0.08 – 0.42 | 0% | **0.003** |
| Sponsored by manufacturer of comparator antipsychotic (N=4, n=490) | 0.04 | -0.14 – 0.21 | 0% | 0.695 |
| Sponsored by both manufacturers of clozapine and comparator antipsychotic (N=2, n=197) | 0.04 | -0.29 – 0.37 | 21% | 0.803 |
| # 2.7 Comparison with individual antipsychotics |  |  |  |  |
| vs. chlorpromazine (N=2, n=50) | 0.12 | -0.45 – 0.69 | 0% | 0.677 |
| vs. haloperidol (N=4, n=368) | 0.25 | 0.04 – 0.45 | 0% | **0.020** |
| vs. olanzapine (N=6, n=466) | 0.06 | -0.12 – 0.24 | 0% | 0.531 |
| vs. risperidone (N=3, n=366) | 0.09 | -0.27 – 0.45 | 50% | 0.627 |
| ***Negative symptoms*** |  |  |  |  |
| #3 Original analysis, non-TRS studies (N=18, n=1,651) | 0.07 | -0.05 – 0.19 | 24% | 0.262 |
| #3.1 Excluding child/adolescent patients (N=16, n=1,587) | 0.05 | -0.08 – 0.17 | 25% | 0.463 |
| #3.2 Excluding studies with crossover design (N=16, n=1,555) | 0.07 | -0.06 – 0.20 | 28% | 0.293 |
| #3.3 Excluding studies with imputed SD of change values (N=9, n=1,200) | 0.05 | -0.12 – 0.22 | 48% | 0.575 |
| #3.4 Excluding studies published before 1988 (N=17, n=1,633) | 0.07 | -0.05 – 0.19 | 25% | 0.284 |
| # 3.5 Disaggregating studies including both TRS and treatment-intolerant patients from non-TRS studies |  |  |  |  |
| Studies including both TRS and treatment-intolerant patients (N=11, n=1,277) | 0.07 | -0.08 – 0.22 | 41% | 0.378 |
| Other non-TRS studies (N=7, n=374) | 0.06 | -0.15 – 0.26 | 0% | 0.594 |
| # 3.6 Categorisation based on industry sponsorship |  |  |  |  |
| No industry sponsorship or sponsorship status unreported (N=6, n=230) | 0.23 | -0.03 – 0.50 | 0% | 0.087 |
| Sponsored by manufacturer of clozapine (N=5, n=711) | 0.21 | 0.05 – 0.36 | 5% | **0.009** |
| Sponsored by manufacturer of comparator antipsychotic (N=6, n=624) | -0.13 | -0.28 – 0.03 | 0% | 0.114 |
| Sponsored by both manufacturers of clozapine and comparator antipsychotic (N=1, n=86) | NA | | | |
| # 3.7 Comparison with individual antipsychotics |  |  |  |  |
| vs. chlorpromazine (N=3, n=210) | 0.04 | -0.23 – 0.32 | 0% | 0.749 |
| vs. haloperidol (N=4, n=345) | 0.24 | -0.01 – 0.49 | 11% | 0.060 |
| vs. olanzapine (N=6, n=518) | -0.03 | -0.20 – 0.14 | 0% | 0.729 |
| vs. risperidone (N=3, n=366) | 0.20 | -0.01 – 0.40 | 0% | 0.061 |

SMD values are shown as effect sizes, with values larger (or smaller) than 0 representing greater (or less) improvement of symptoms in patients receiving clozapine compared to other antipsychotics. *P*-values of <0.05 are shown in bold. Abbreviations: CI, confidence interval; NA, not applicable; SD, standard deviation; SMD, standardised mean difference; TRS, treatment resistant schizophrenia.
