# Supplementary material for: Heterogeneity and efficacy of antipsychotic treatment for schizophrenia with or without treatment resistance: a meta-analysis
Source: Neuropsychopharmacology. 2019 Nov 25;45(4):622–31. doi: 10.1038/s41386-019-0577-3 (PMC7021799; doi:10.1038/s41386-019-0577-3)
Supplement: Supplementary file 2 — Supplementary Figures 1-4 [file 41386_2019_577_MOESM2_ESM.doc]

**Figure S1.** PRISMA flowchart of literature search and study selection

Records identified in electronic search (n=3,886)

(Embase, MEDLINE, and PsycINFO combined in Ovid 3,026; Cochrane CENTRAL 706; Clinicaltrials.gov 154)

**Screening**

**Included**

**Eligibility**

**Identification**

Records after duplicates removed (n=2,510)

Titles and record types screened (n=2,510)

Records excluded (n=2,360)

Full-text assessed for eligibility (n=99)

Records excluded for the following reasons:

Overlap with included primary publication (n=35)

Primary publication reported in non-English language (n=14)

Not a double-blind randomised controlled trial (n=3)

Data not published (n=3)

Published, but no extractable data (n=2)

Additional review article (n=1)

Unclear diagnosis of included participants (n=1)

Primary publication not identified (n=1)

Studies included in the meta-analysis

(n=39)

(Strictly-defined TRS studies 10; Other non-refractory schizophrenia studies 29)

Primary publications of eligible studies (n=39)

Additional publications identified by hand-search

of reference lists (n=0)

Abstracts screened (n=150)

Records excluded (n=51)

Abbreviations: TRS, treatment-resistant schizophrenia

**Figure S2.** Scatterplots showing relationship between adjusted mean change and standard deviation of change in symptom scores for studies included in the meta-analysis of variance. Significant positive correlations were evident for total symptoms (r=0.418, *p*=0.017), positive symptoms (r=0.842, *p<*0.001) and negative symptoms (r=0.794, *p<*0.001). These results indicate mean scaling of variability, and provide rationale for using the coefficient of variation ratio (CVR) as a complementary measure in the meta-analysis of variability.

S2a. Total symptoms (Spearman’s rank correlation coefficient r=0.418, ***p*=0.017**)


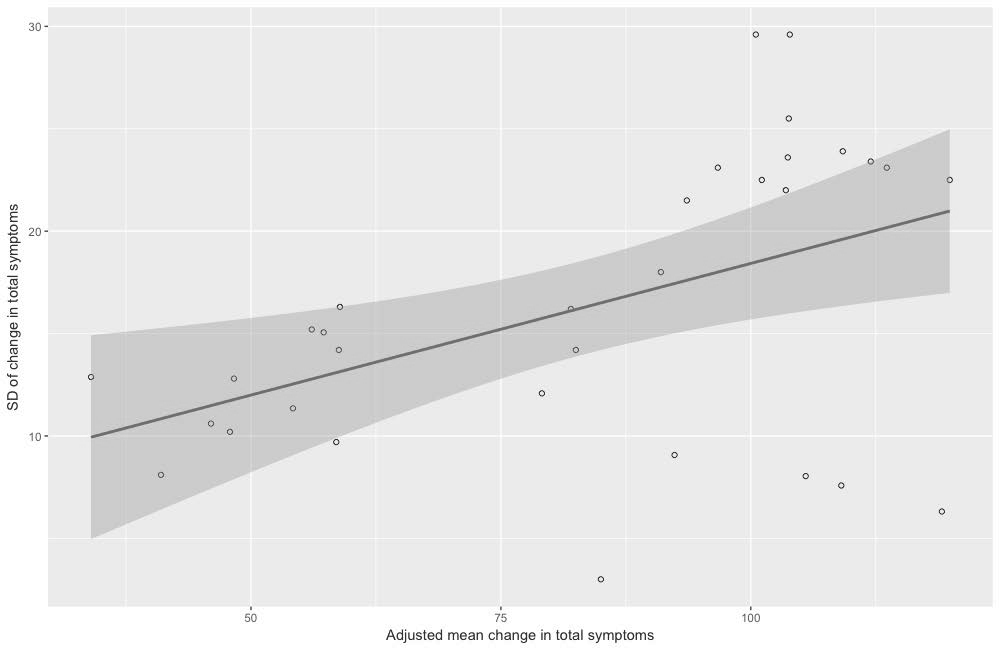


S2b. Positive symptoms (Spearman’s rank correlation coefficient r=0.842, ***p<*0.001**)


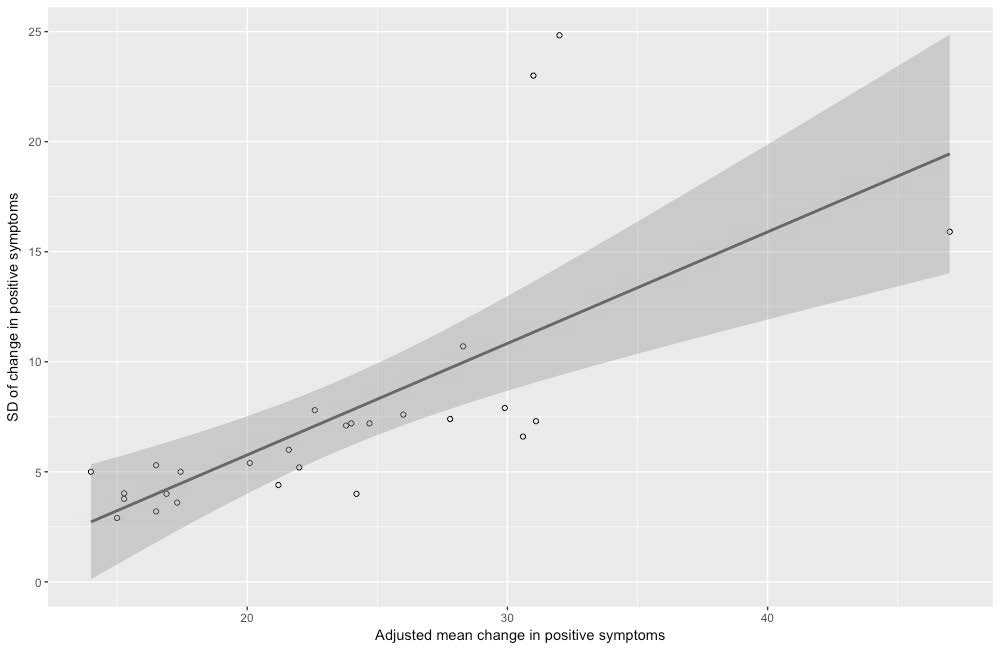


S2c. Negative symptoms (Spearman’s rank correlation coefficient r=0.794, ***p<*0.001**)


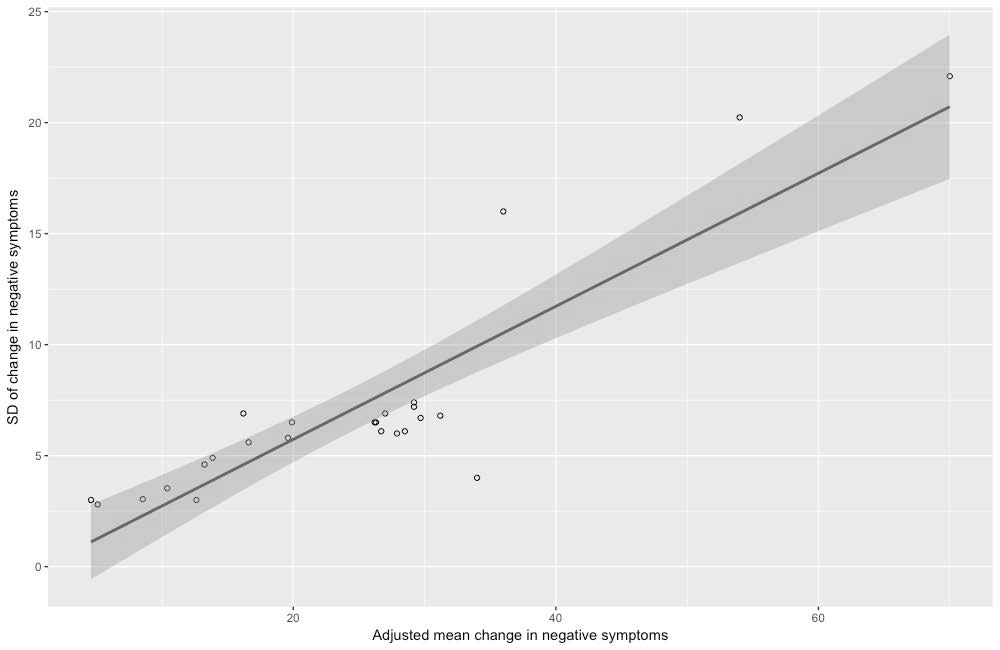


Scatterplots show data points for clozapine and other antipsychotic groups combined. *P*-values of <0.05 are shown in bold.

**Figure S3.** Forest plot showing effect sizes of VR/CVR for change in positive symptoms. In studies of strictly-defined treatment resistant schizophrenia (TRS), there is no significant alteration in the summary variability ratio (VR=1.61, *p*=0.185) or the summary correlation of variation ratio (CVR=1.21, *p*=0.334), indicating that the variability in response to treatment is the same in patients receiving clozapine as other antipsychotics. In studies of other non-refractory schizophrenia (non-TRS), the summary variability ratio indicates smaller variability in response to treatment with clozapine (VR=0.92, *p*=0.031); however, this was not evident when accounting for the difference in means (CVR=0.90, *p*=0.170).

S3a. Positive symptoms, Variability Ratios (VR)


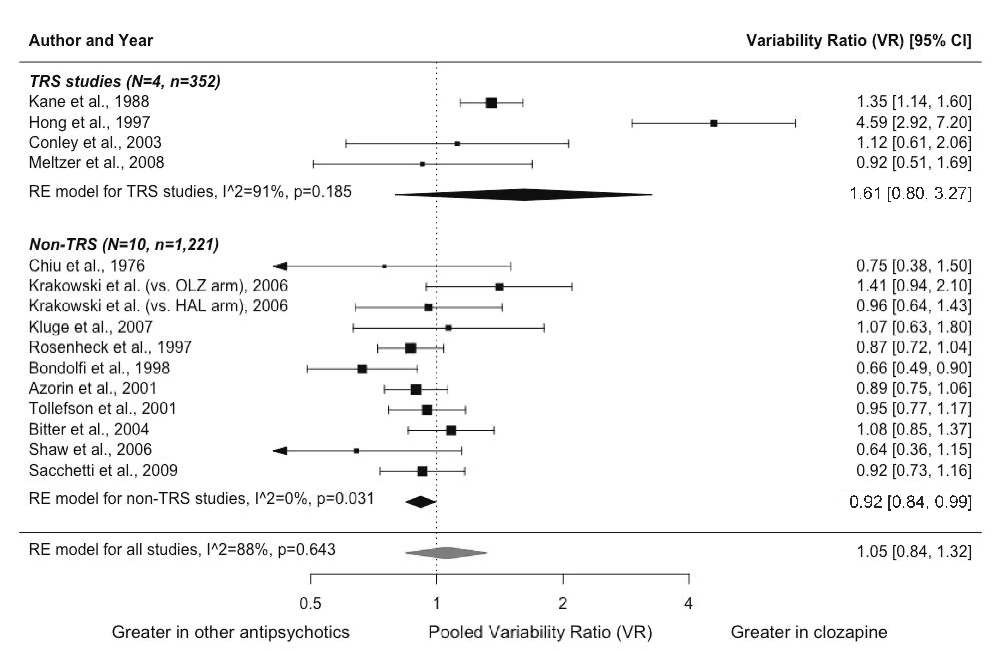


S3b. Positive symptoms, Coefficient of Variation Ratios (CVR)


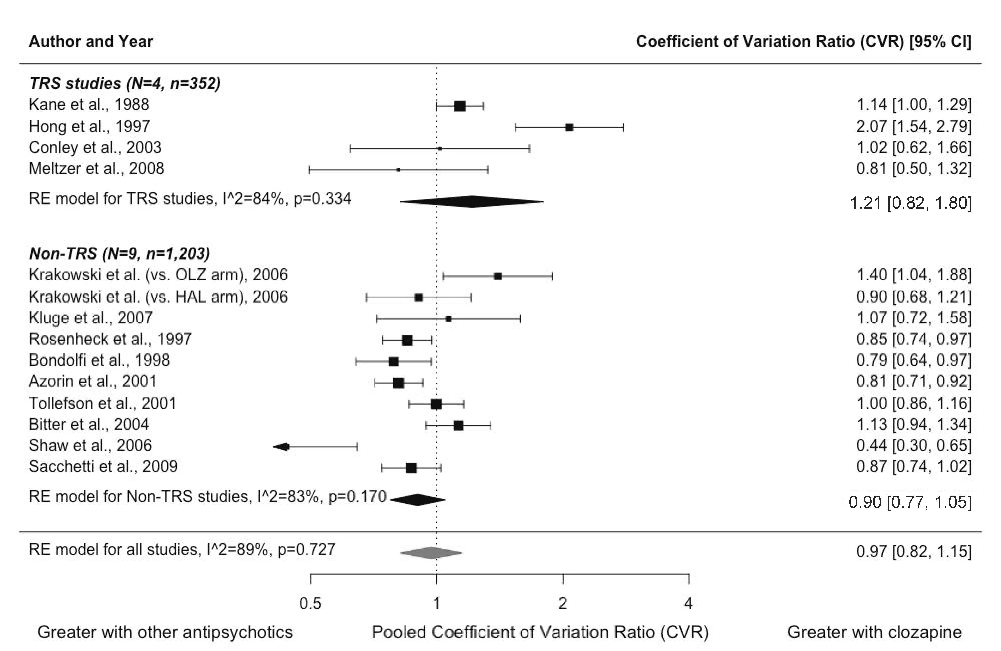


Abbreviations: CI, confidence interval; HAL, haloperidol; OLZ, olanzapine; RE, random effects.

**Figure S4.** Forest plot showing effect sizes of VR/CVR for change in negative symptoms. In studies of strictly-defined treatment resistant schizophrenia (TRS), there is no significant alteration in the summary variability ratio (VR=1.61, *p*=0.122) or the summary correlation of variation ratio (CVR=1.88, *p*=0.074), indicating that the variability in response to treatment is the same in patients receiving clozapine as other antipsychotics. Similar results applied for studies of other non-refractory schizophrenia (non-TRS) (VR=1.02, *p*=0.620; CVR=1.00, *p*=0.950).

S4a. Negative symptoms, Variability Ratios (VR)


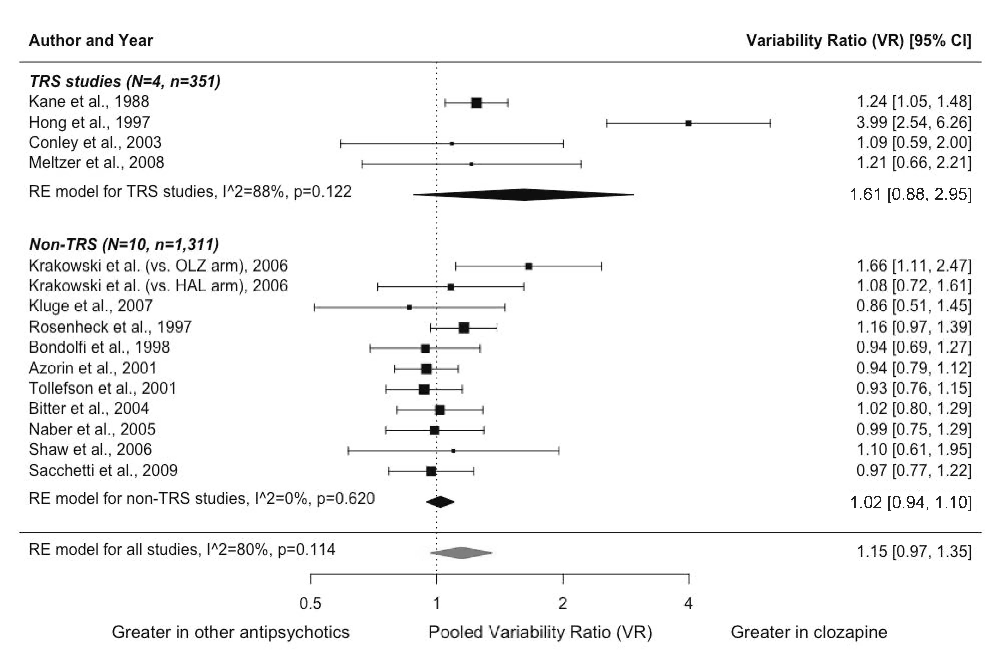


S4b. Negative symptoms, Coefficient of Variation Ratios (CVR)


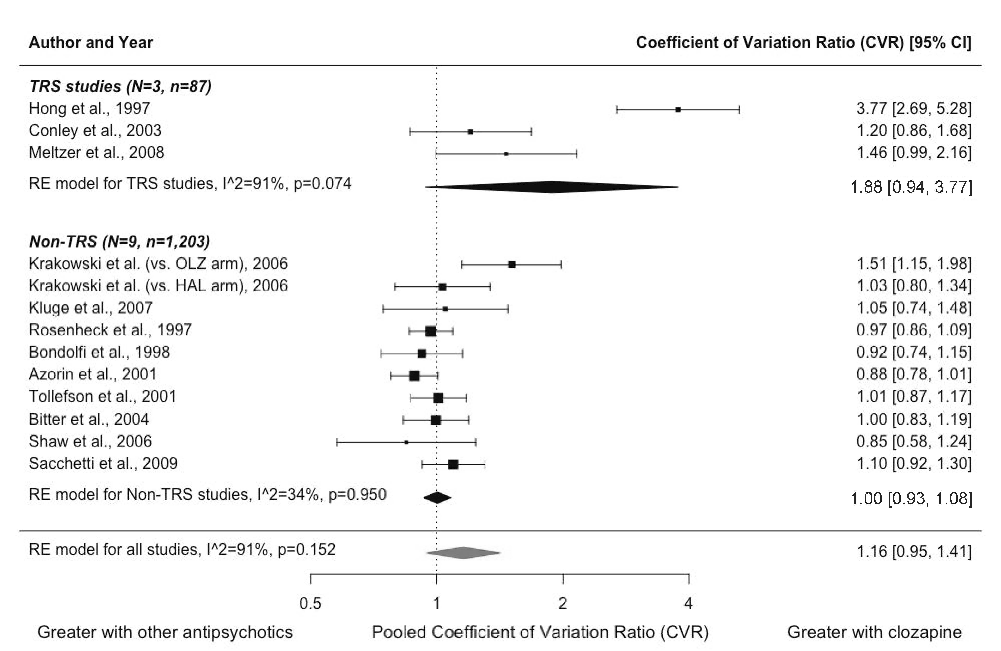


Abbreviations: CI, confidence interval; HAL, haloperidol; OLZ, olanzapine; RE, random effects.
