# Supplementary material for: Heterogeneity and efficacy of antipsychotic treatment for schizophrenia with or without treatment resistance: a meta-analysis
Source: Neuropsychopharmacology. 2019 Nov 25;45(4):622–31. doi: 10.1038/s41386-019-0577-3 (PMC7021799; doi:10.1038/s41386-019-0577-3)

**Figure S5.** Meta-regression regarding VR/CVR of change in total symptoms against the number of items fulfilled in the TRRIP consensus criteria. These results indicate that TRS studies, and in particular rigorously defined TRS is associated with greater variability in response to treatment with clozapine relative to other antipsychotics (VR: z=2.790, *p*=0.005; CVR: z=2.245, *p*=0.025).

S5a. Total symptoms, Variability Ratios (VR), z=2.790, ***p*=0.005**


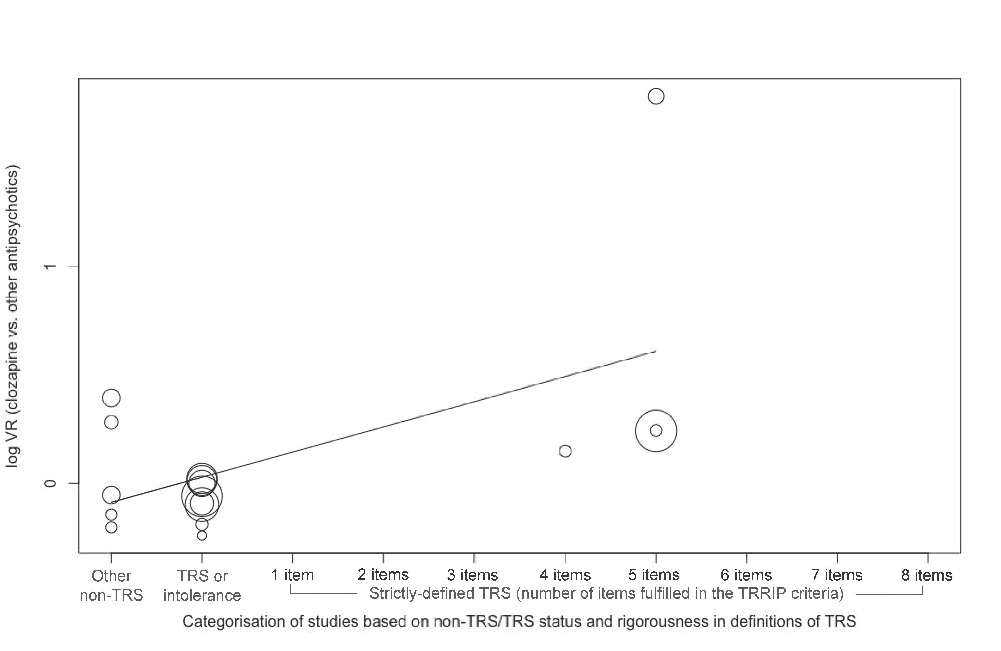


S5b. Total symptoms, Coefficient of Variation Ratios (CVR), z=2.245, ***p*=0.025**


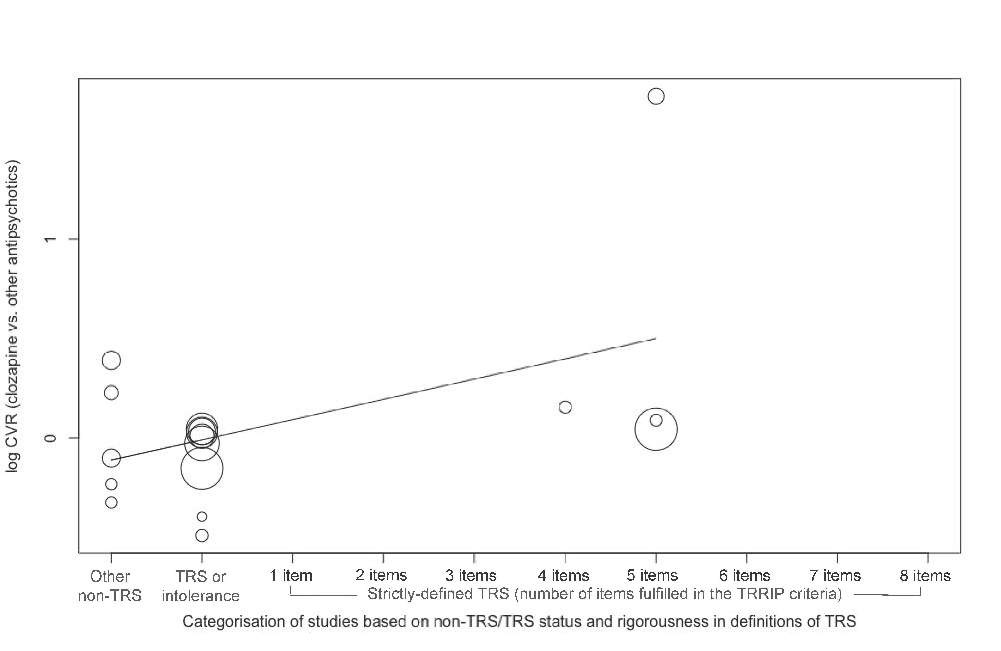


**Figure S6.** Forest plot showing SMD for change in positive symptoms in TRS and non-TRS studies. The standardised mean difference (SMD) in studies of strictly-defined treatment resistant schizophrenia (TRS) indicate that clozapine is more effective in improving positive symptoms compared to other antipsychotics (*g*=0.32, *p*=0.003). The same finding applied to studies of other non-refractory schizophrenia (non-TRS) (*g*=0.15, *p*=0.006).

Positive symptoms


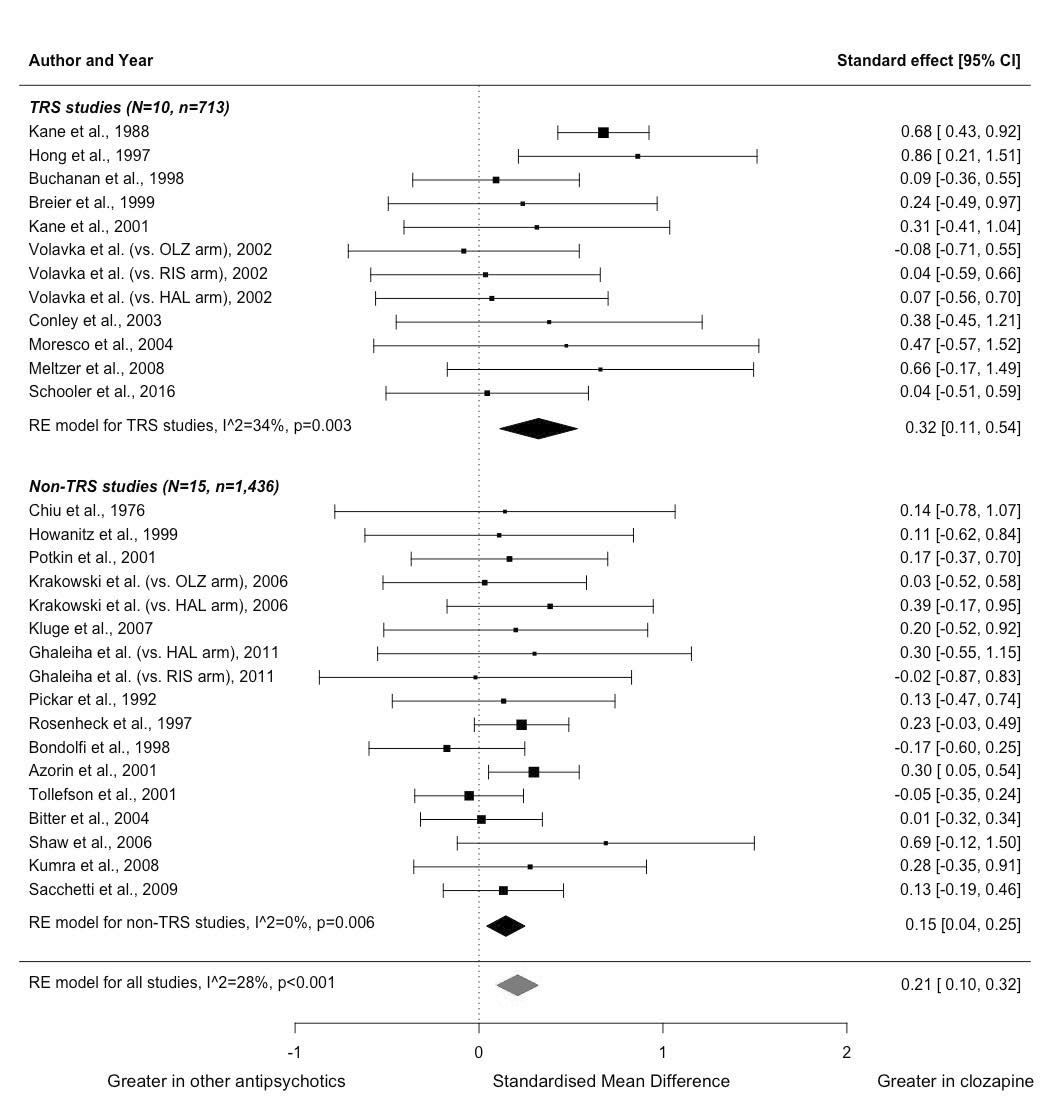


Abbreviations: CI, confidence interval; HAL, haloperidol; OLZ, olanzapine; RE, random effects; RIS, risperidone.

**Figure S7.** Forest plot showing SMD for change in negative symptoms in TRS and non-TRS studies. The standardised mean difference (SMD) in studies of strictly-defined treatment resistant schizophrenia (TRS) indicate that clozapine is not superior to other antipsychotics in improving negative symptoms (*g*=0.22, *p*=0.135). This finding also applied to studies of other non-refractory schizophrenia (non-TRS) (*g*=0.07, *p*=0.262).

Negative symptoms


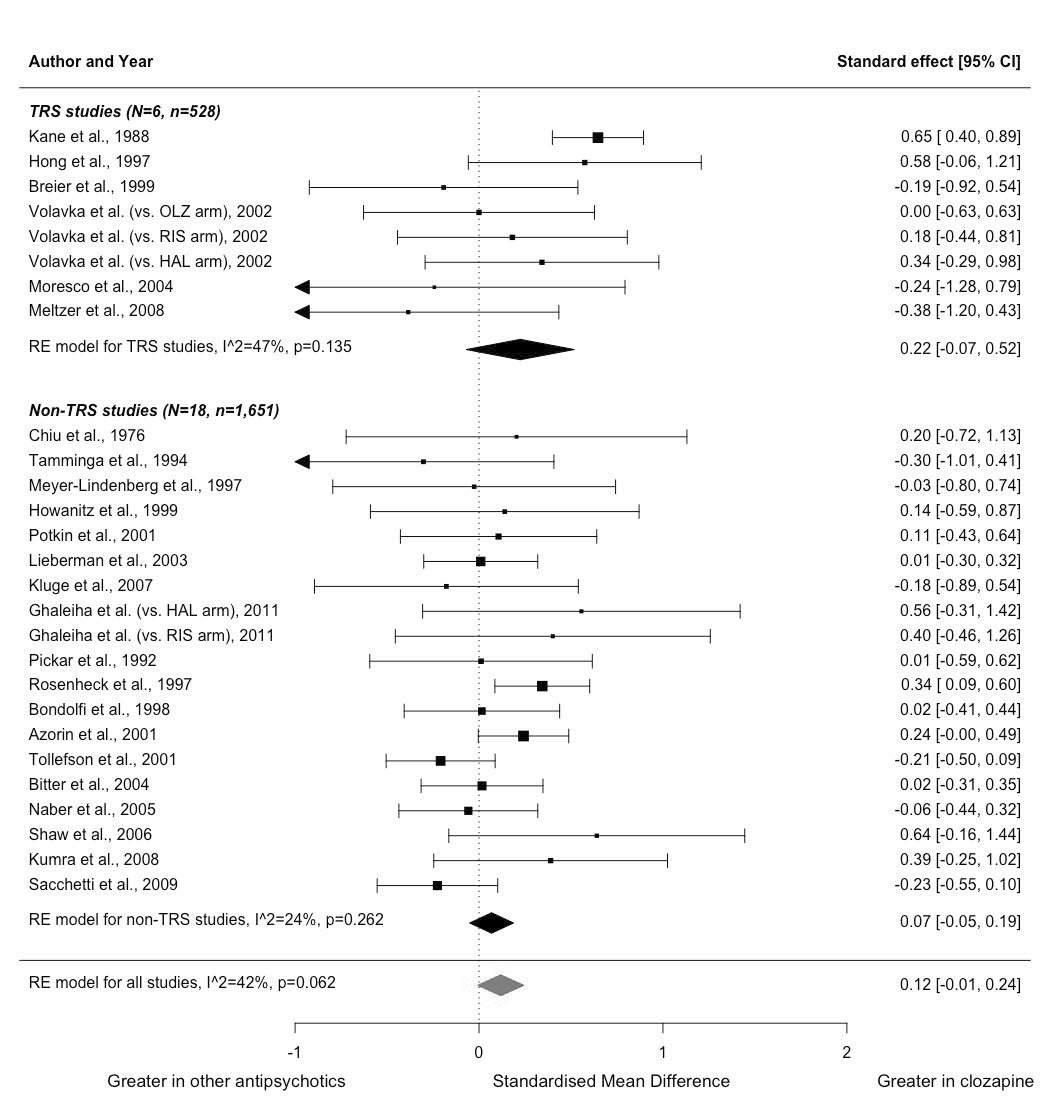


Abbreviations: CI, confidence interval; HAL, haloperidol; OLZ, olanzapine; RE, random effects; RIS, risperidone.

**Figure S8.** Funnel plots for SMD of change in total symptoms. Visual inspection of funnel plots and regression tests did not indicate publication bias for standardised mean difference (SMD) in studies of strictly-defined treatment resistant schizophrenia (TRS) (z=-0.734, *p*=0.463). However, there was indication of publication bias in studies of other non-refractory schizophrenia (non-TRS) (z=2.557, ***p*=0.011**).

S8a. Total symptoms, TRS studies, regression test: z=-0.734, *p*=0.463


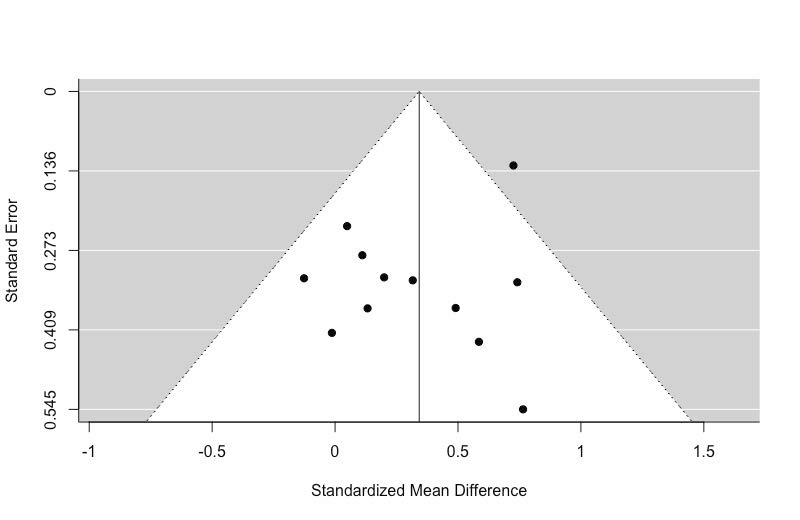


S8b. Total symptoms, Non-TRS studies, regression test: z=2.557, ***p*=0.011**


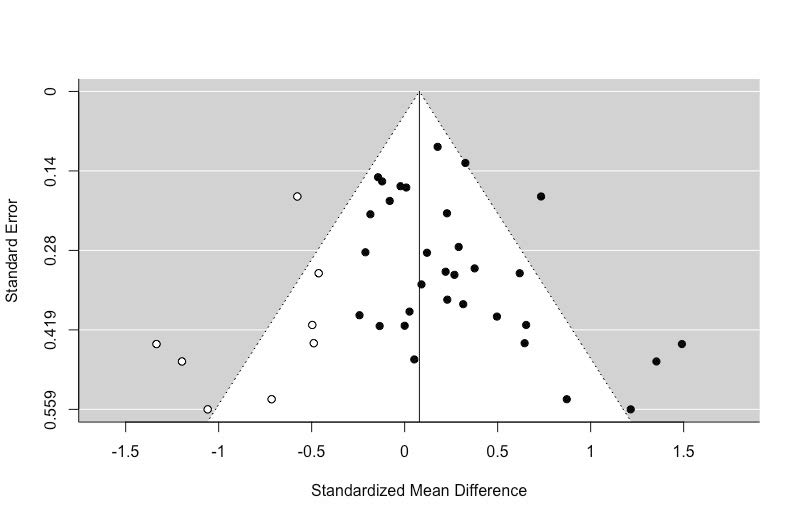

Supplement: Supplementary file 3 — Supplementary Figures 5-8 [file 41386_2019_577_MOESM3_ESM.doc]
