# Supplementary material for: Heterogeneity and efficacy of antipsychotic treatment for schizophrenia with or without treatment resistance: a meta-analysis
Source: Neuropsychopharmacology. 2019 Nov 25;45(4):622–31. doi: 10.1038/s41386-019-0577-3 (PMC7021799; doi:10.1038/s41386-019-0577-3)

**Figure S9.** Meta-regression regarding SMD of change in total symptoms in all studies. Data points include both studies of strictly-defined treatment resistant schizophrenia (TRS) and other non-refractory schizophrenia (non-TRS).

S9a. Meta-regression of change in total symptoms against the number of items fulfilled in the TRRIP consensus criteria. The results show that TRS studies, and in particular rigorously defined TRS is not a significant moderator of SMD for total symptoms, z=0.911, *p*=0.362.

**
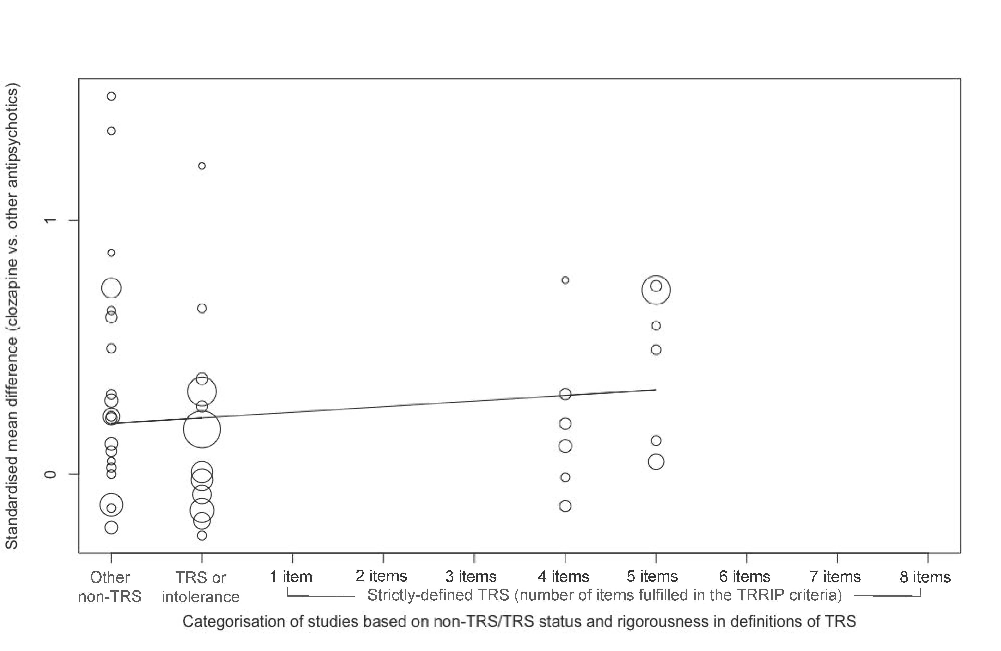
**

S9b. Meta-regression of change in total symptoms against baseline symptom severity (total PANSS equivalent). The results show that baseline symptom severity is not a significant moderator of SMD for total symptoms, z=1.679, *p*=0.093.

**
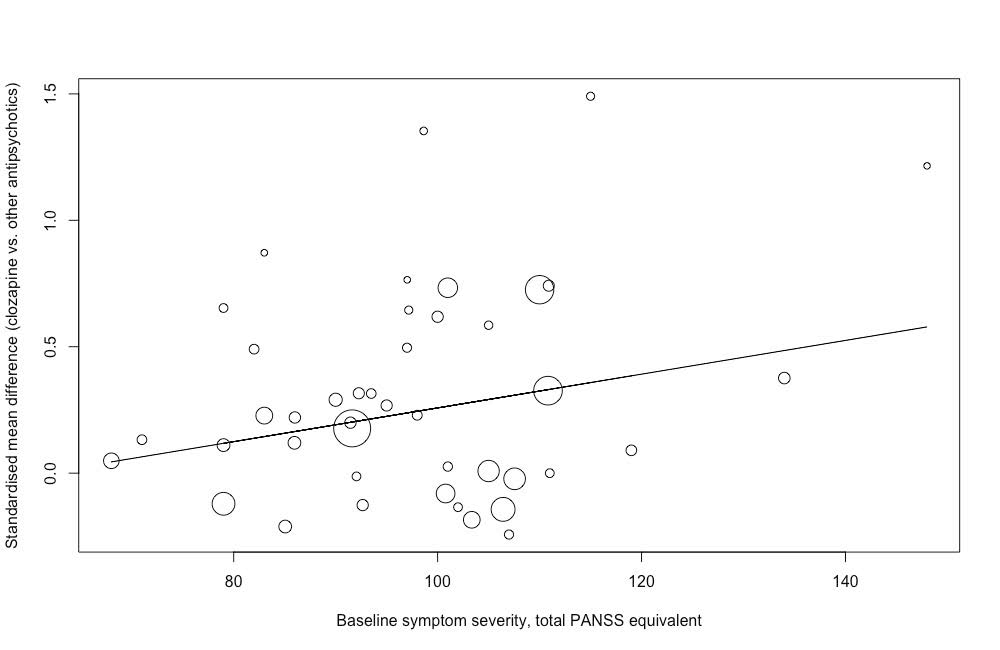
**

S9c. Meta-regression of change in total symptoms against mean dose of clozapine (mg/day). The results show that a greater mean daily dose of clozapine is associated with greater improvement of total symptoms with clozapine relative to other antipsychotics, z=1.959, ***p*=0.050**.


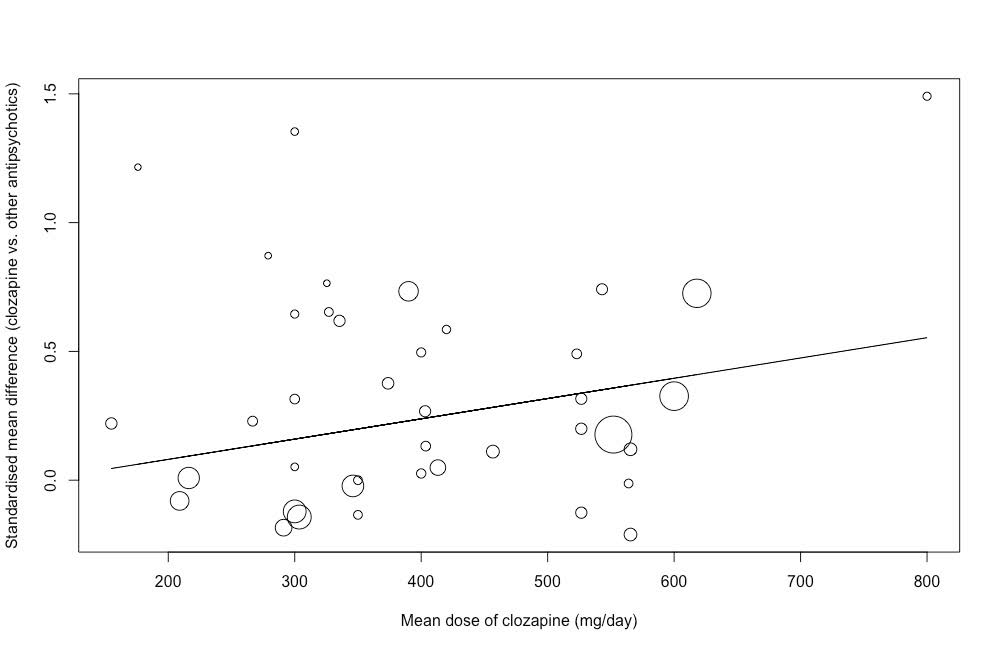


S9d. Meta-regression of change in total symptoms against difference between chlorpromazine equivalent (CPZE) dose of comparator antipsychotic and clozapine (mg/day). Greater values on the x-axis indicate greater CPZE equivalent doses of comparator antipsychotics relative to clozapine. The results show that difference between CPZE dose of comparator antipsychotic and clozapine is not a significant moderator of SMD for total symptoms, z=1.733, *p*=0.083.


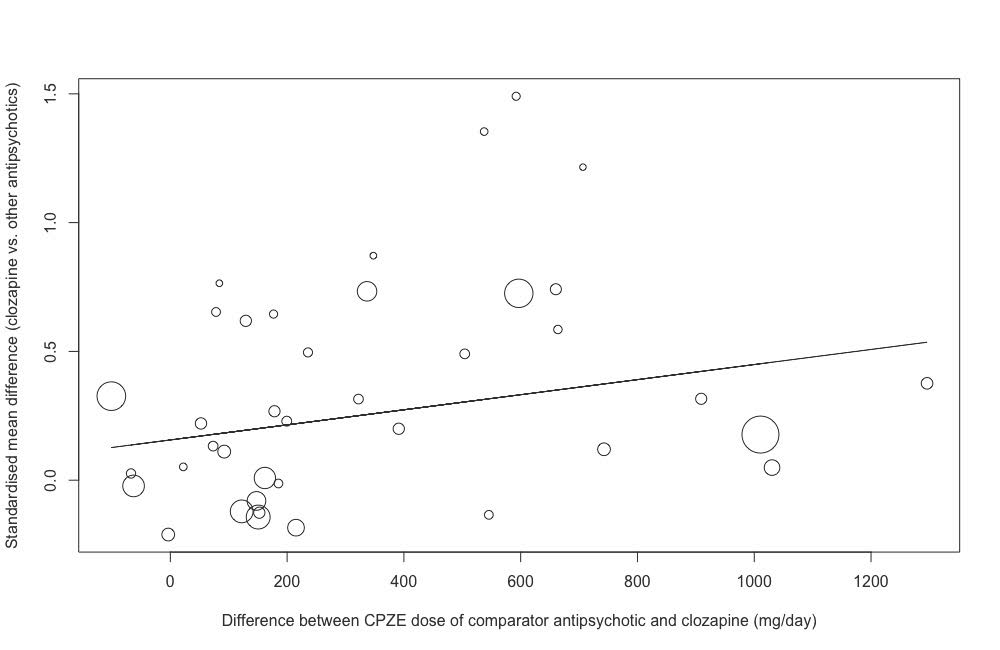


S9e. Meta-regression of change in total symptoms against duration of intervention (weeks). The results show that longer duration of double-blind intervention is associated with less improvement of total symptoms with clozapine relative to other antipsychotics, z=-2.169, ***p*=0.030.**

**
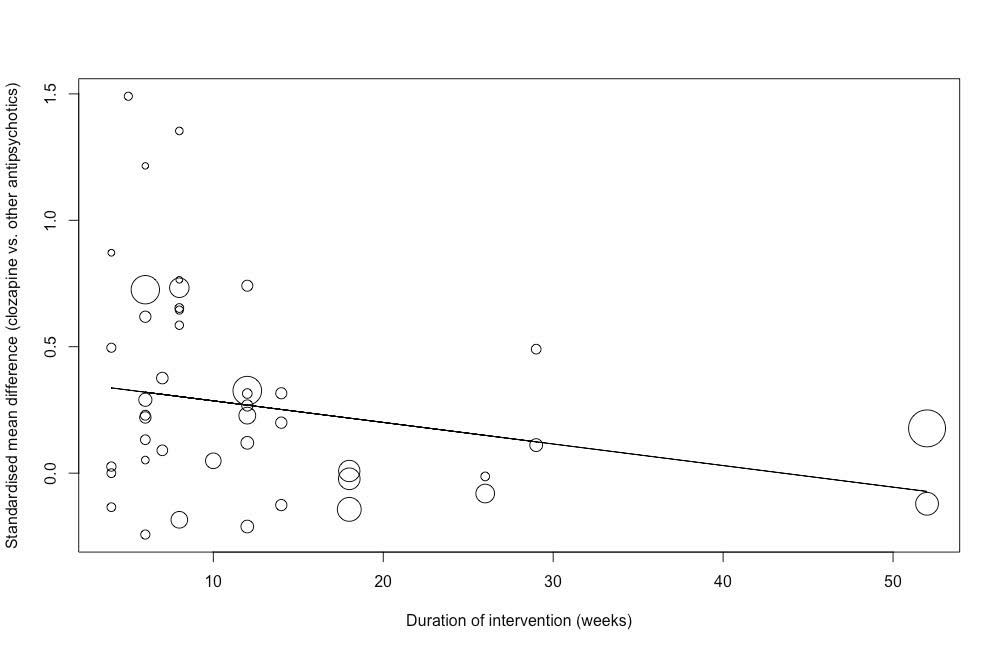
**

Abbreviations: PANSS, Positive and Negative Syndrome Scale

**Figure S10.** Meta-regression regarding SMD of change in positive symptoms in all studies. Data points include both studies of strictly-defined treatment resistant schizophrenia (TRS) and other non-refractory schizophrenia (non-TRS).

S10a. Meta-regression of change in positive symptoms against the number of items fulfilled in the TRRIP consensus criteria. The results show that TRS studies, and in particular more rigorously-defined TRS is associated with greater improvement in positive symptoms with clozapine relative to other antipsychotics, z=2.168, ***p*=0.030**.

**
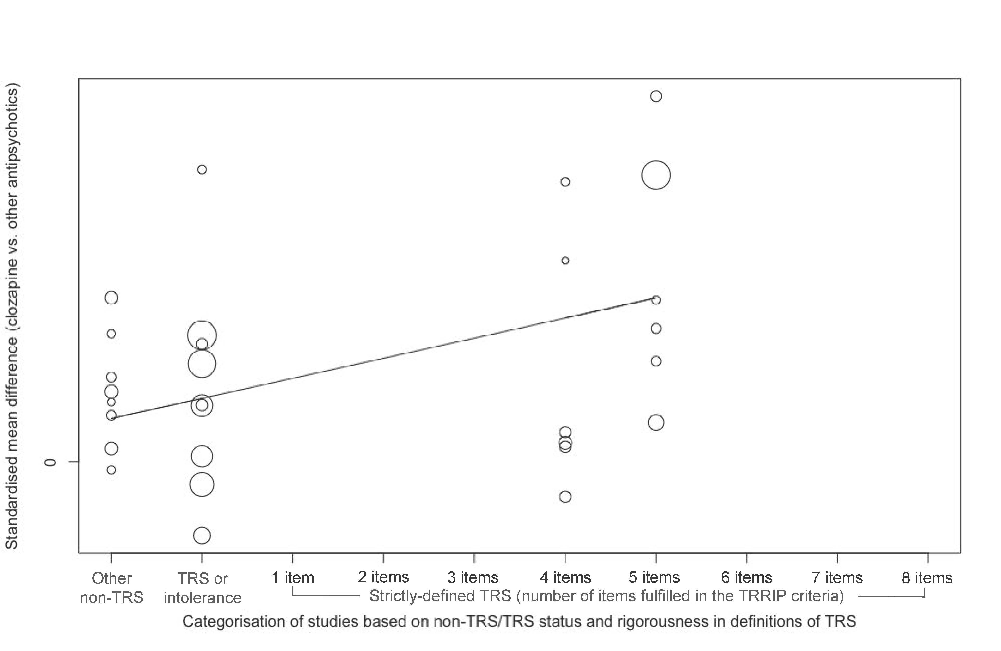
**

S10b. Meta-regression of change in positive symptoms against baseline symptom severity (total PANSS equivalent). The results show that baseline symptom severity is not a significant moderator of SMD for positive symptoms, z=0.570, *p*=0.569.


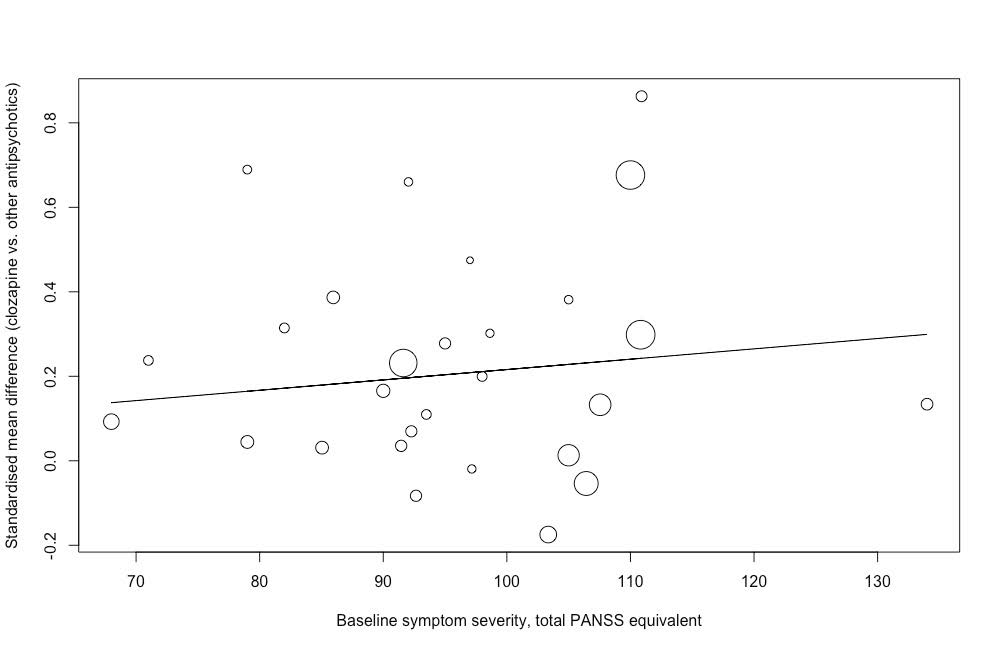


S10c. Meta-regression of change in positive symptoms against mean dose of clozapine (mg/day). The results show that higher mean dose of clozapine is associated with greater improvement of positive symptoms with clozapine relative to other antipsychotics, z=3.403, ***p*<0.001**.


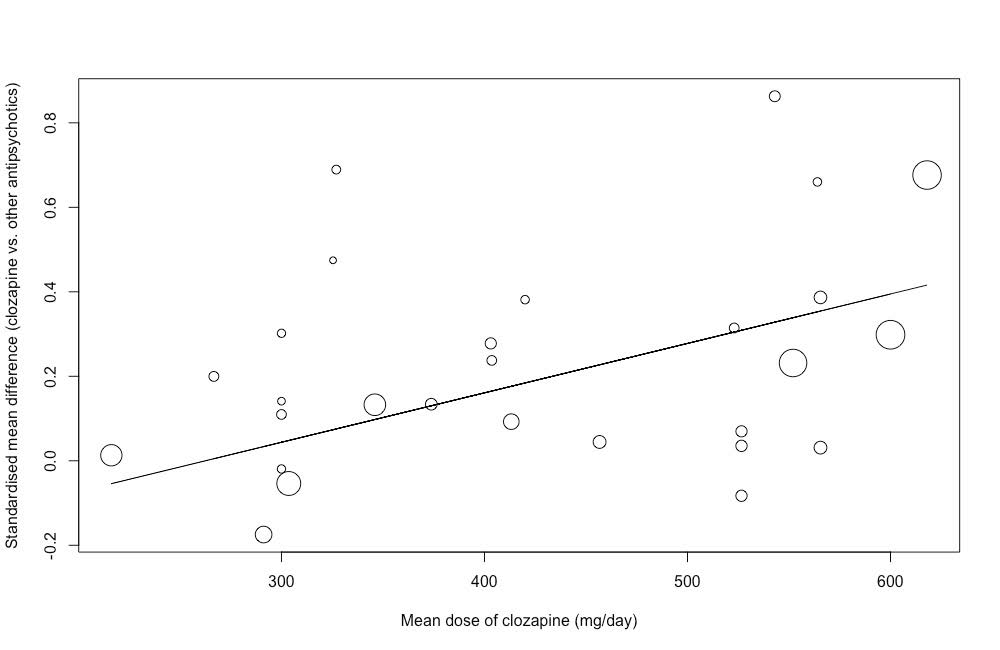


S10d. Meta-regression of change in positive symptoms against difference between chlorpromazine equivalent (CPZE) dose of comparator antipsychotic and clozapine (mg/day). Greater values on the x-axis indicate greater CPZE equivalent doses of comparator antipsychotics relative to clozapine. The results show that difference between CPZE dose of comparator antipsychotic and clozapine is not a significant moderator of SMD for positive symptoms, z=0.911, *p*=0.362.


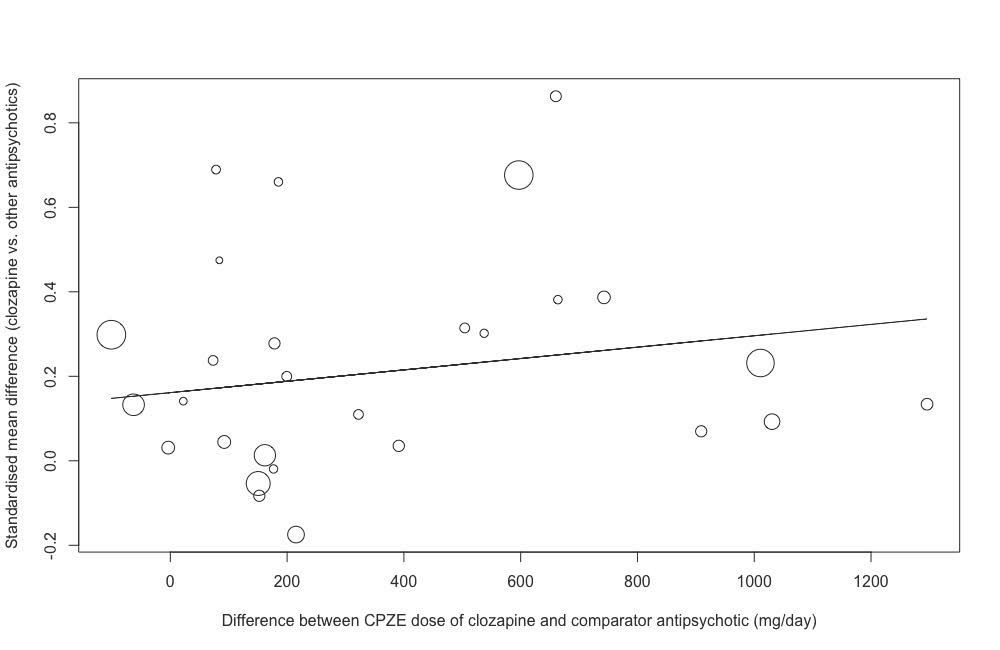


S10e. Meta-regression of change in positive symptoms against duration of intervention (weeks). The results show that duration of double-blind intervention is not a significant moderator of SMD for positive symptoms, z=-0.431, *p*=0.666**.**


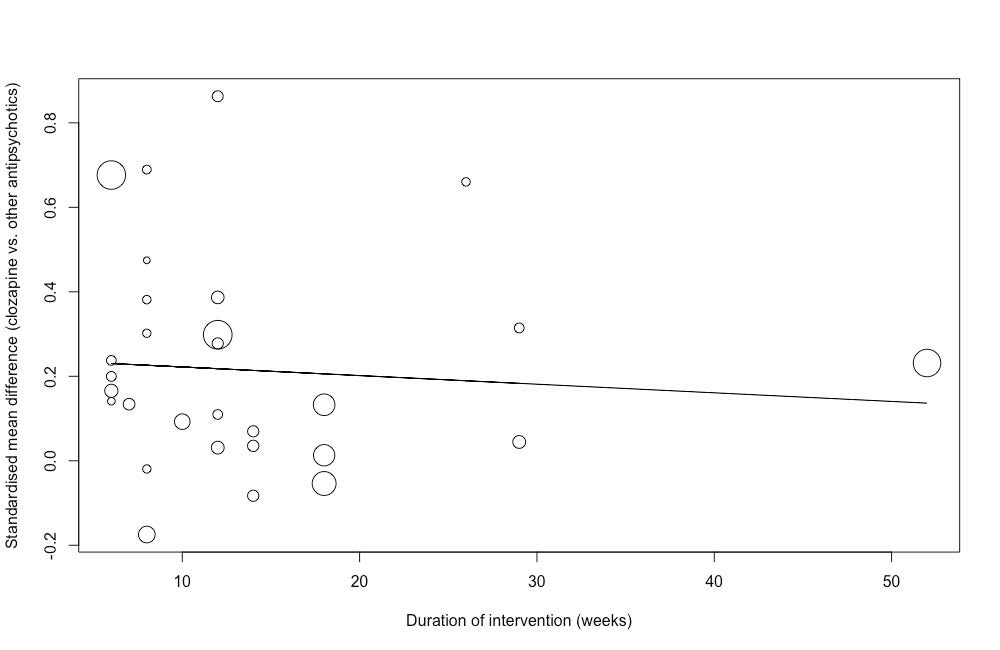


Abbreviations: PANSS, Positive and Negative Syndrome Scale

**Figure S11.** Meta-regression regarding SMD of change in negative symptoms in all studies. Data points include both studies of strictly-defined treatment resistant schizophrenia (TRS) and other non-refractory schizophrenia (non-TRS).

S11a. Meta-regression of change in negative symptoms against the number of items fulfilled in the TRRIP consensus criteria. The results show that TRS studies, and in particular more rigorously-defined TRS is not a significant moderator of SMD for negative symptoms , z=1.863, *p*=0.063.


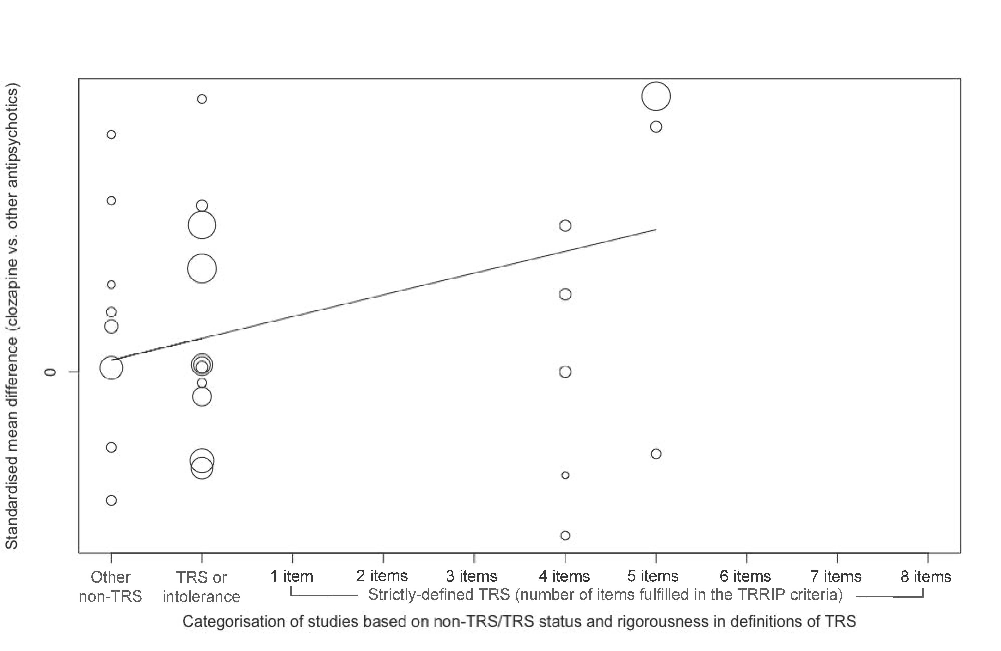


S11b. Meta-regression of change in negative symptoms against baseline symptom severity (total PANSS equivalent). The results show that baseline symptom severity is not a significant moderator of SMD for negative symptoms, z=0.200, *p*=0.842.

**
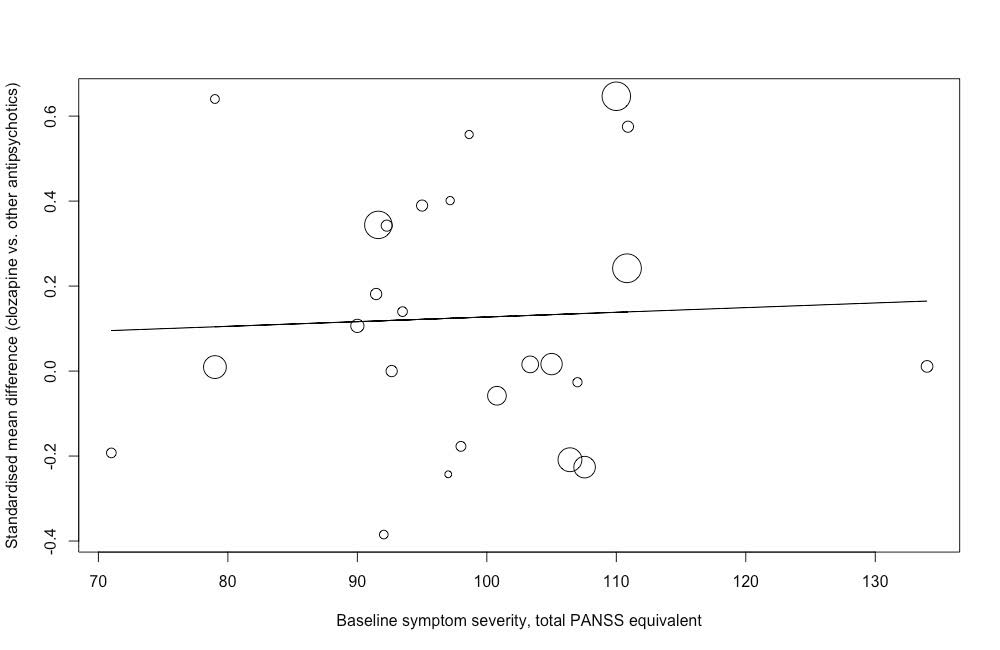
**

S11c. Meta-regression of change in negative symptoms against mean dose of clozapine (mg/day). The results show that higher mean dose of clozapine is associated with greater improvement of negative symptoms with clozapine relative to other antipsychotics, z=3.797, ***p*<0.001**.


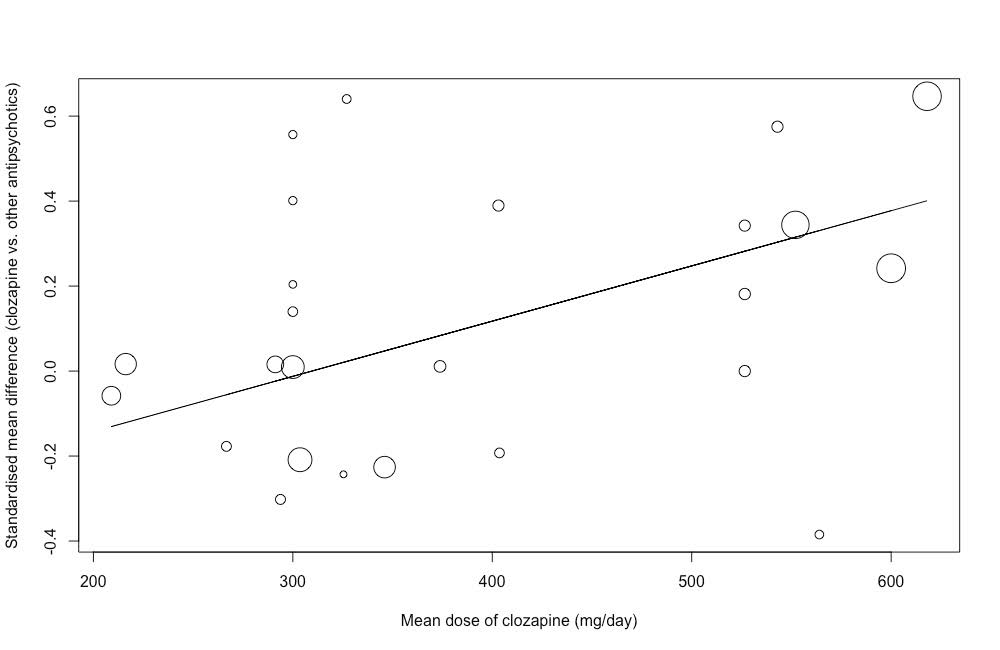


S11d. Meta-regression of change in negative symptoms against difference between chlorpromazine equivalent (CPZE) dose of comparator antipsychotic and clozapine (mg/day). Greater values on the x-axis indicate greater CPZE equivalent doses of comparator antipsychotics relative to clozapine. The results show that difference between CPZE dose of comparator antipsychotic and clozapine is not a significant moderator of SMD for negative symptoms, z=1.444, *p*=0.149.


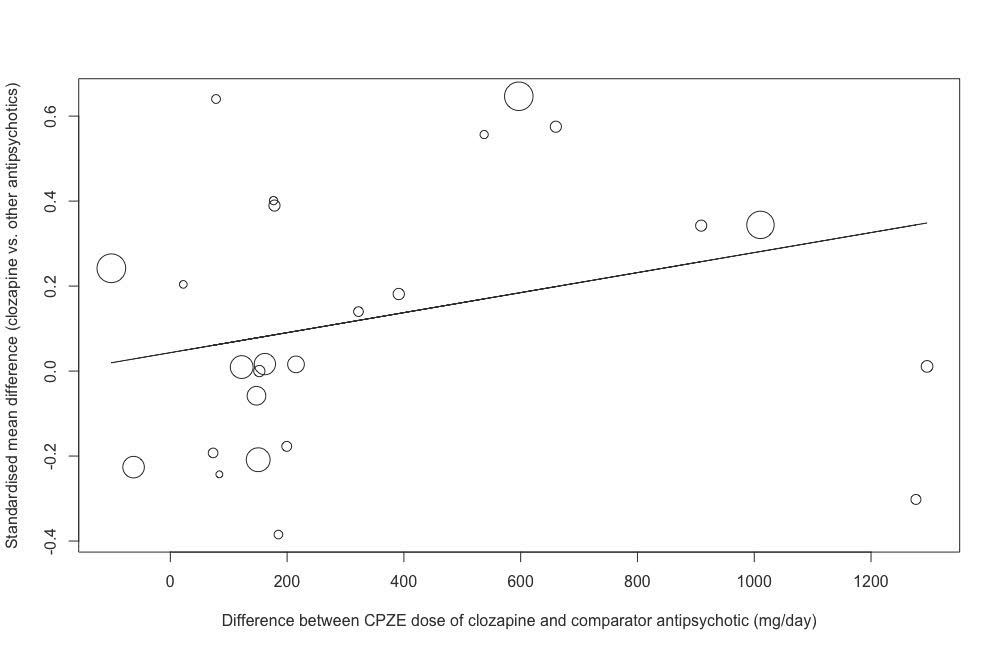


S11e. Meta-regression of change in negative symptoms against duration of intervention (weeks). The results show that duration of double-blind intervention is not a significant moderator of SMD for negative symptoms, z=-0.757, *p*=0.449**.**


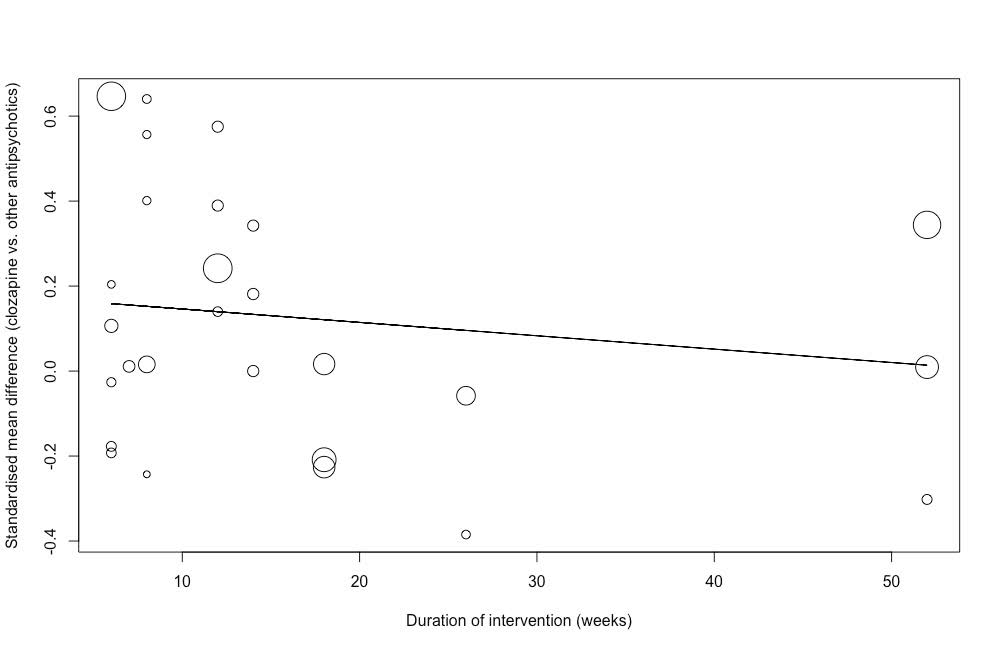


Abbreviations: PANSS, Positive and Negative Syndrome Scale

**Figure S12.** *Post hoc* meta-regression of change in total symptoms against mean duration of illness in non-TRS studies. The results show that duration of illness in study samples is not a significant moderator of effect size, z=-0.158, *p*=0.874.


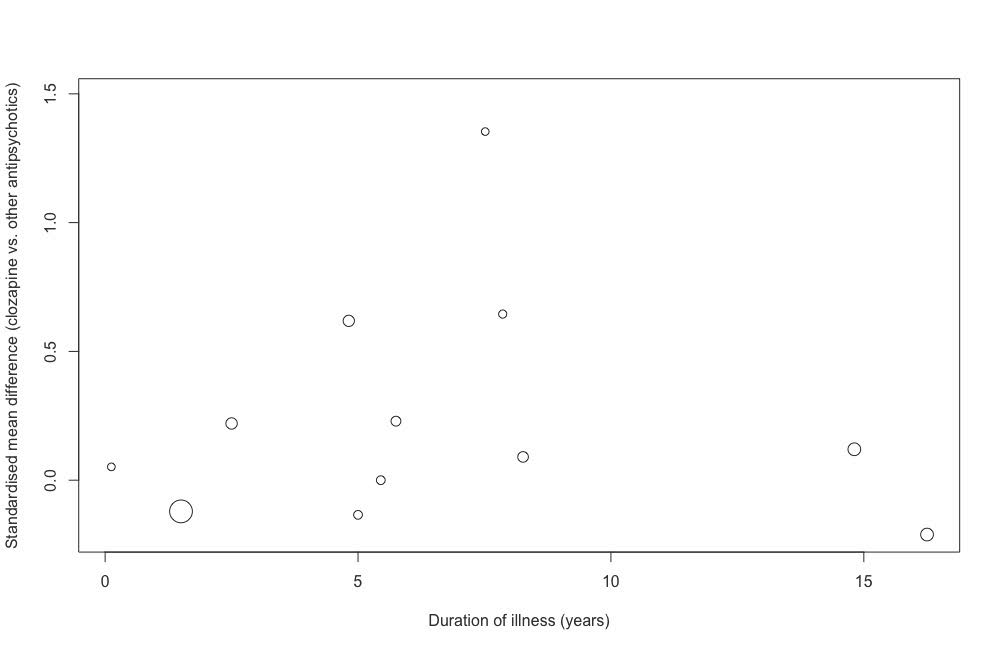

Supplement: Supplementary file 4 — Supplementary Figures 9-12 [file 41386_2019_577_MOESM4_ESM.docx]
