# Supplementary material for: Heterogeneity and efficacy of antipsychotic treatment for schizophrenia with or without treatment resistance: a meta-analysis
Source: Neuropsychopharmacology. 2019 Nov 25;45(4):622–31. doi: 10.1038/s41386-019-0577-3 (PMC7021799; doi:10.1038/s41386-019-0577-3)
Supplement: Supplementary file 5 — Supplementary Materials and Methods [file 41386_2019_577_MOESM5_ESM.pdf]

Supplementary Materials and Methods. Study protocol

Variance in response to clozapine vs. all other antipsychotics in patients with schizophrenia: A systematic review and meta-analysis

Protocol Date: 19 January 2018

Yuya Mizuno, MRCPsych PhD[1,2]
Robert A. McCutcheon, MRCPsych[1,3,4]
Stefan P. Brugger, MBBS, MSc[3,4,5]
Oliver D. Howes, MRCPsych PhD[1,3,4]

[1] Department of Psychosis Studies, Institute of Psychiatry, Psychology & Neuroscience, King's College London, London, UK
[2] Department of Neuropsychiatry, Keio University School of Medicine, Tokyo, Japan
[3] Psychiatric Imaging Group, MRC London Institute of Medical Sciences, Hammersmith Hospital, London, UK
[4] Institute of Clinical Sciences, Faculty of Medicine, Imperial College London, London, UK
[5] Division of Psychiatry, University College London, London, UK

TABLE OF CONTENTS

	Page
**I. INTRODUCTION**	
Rationale	3
Objectives	4
**II. METHODS**	
Protocol and registration	5
Systematic review	
Eligibility criteria	5
Information sources	5
Search	5-6
Study selection	6
Data collection process	6
Data items	6
Missing data	6-7
Study categorisation	7
Risk of bias in individual studies	7-8
Meta-analysis	
Summary measures	8-9
Synthesis of results	9
Risk of bias across studies	9
Additional analyses	9-10
**III. TIME TABLE / WORK PLAN**	
Time schedule	11
Dissemination strategy	11
**X. REFERENCES**	12-14

I. Introduction

Rationale

Schizophrenia is a serious mental illness with a lifetime prevalence of approximately 0.7% [1]. It is characterised by psychotic symptoms including delusions and hallucinations, negative symptoms including amotivation and social withdrawal, and cognitive impairment. Schizophrenia is a leading cause of global disease burden [2], and is associated with significant burden on caregivers [3]. Antipsychotic drugs remain the cornerstone of treatment for schizophrenia. However, there is heterogeneity in how patients respond to antipsychotics from early stages of illness [4,5]. About one-third of patients are categorised as having treatment-resistant schizophrenia (TRS) [6], defined as inadequate response to two trials of antipsychotic treatment at adequate dose, duration and adherence [7]. Treatment-resistance is a major challenge to clinical management, and is associated with decreased quality of life, increased medical costs, and increased rates of serious comorbidities [8]. A better understanding regarding the underlying neurobiology of TRS is imperative to improve the use of current treatments and inform development of novel drugs.

Clozapine has established efficacy and is currently the only medication licensed for patients with TRS [9]. Its clinical superiority in TRS compared to chlorpromazine was first shown in the 1980s [10], and subsequently confirmed in landmark clinical trials [11,12]. About 40-60% of patients with TRS respond to clozapine treatment [13-15]. Furthermore, clozapine is associated with lower risks of rehospitalization [16], suicidality [17], and mortality [18] compared to other oral antipsychotics. A recent meta-analysis comparing clozapine with first- and second-generation antipsychotics in TRS showed that clozapine was superior to both categories of drugs in reducing positive symptoms [19], although clozapine's superiority was not supported in a network meta-analysis utilizing both direct and indirect comparisons of antipsychotics [20].

While the overall evidence suggests that clozapine's efficacy in TRS is unique among antipsychotic medications, the neurobiological basis of this differential effect remains elusive. Molecular imaging studies have shown robust evidence for elevated presynaptic dopaminergic function in schizophrenia [21], and all antipsychotics act downstream of this dysfunction by blocking dopamine D2 receptors [22]. In contrast, positron emission tomography (PET) studies focusing on TRS indicate that patients with treatment-resistance have lower presynaptic dopamine function compared to those responding to first-line treatment [23,24]. Thus, TRS may have a distinct neurobiological basis from illness that is responsive to first-line antipsychotics, which is characterised by an absence of hyperdopaminergia. The mechanism which makes clozapine unique in this context remains controversial, although lower affinity to- and faster disassociation from D2 receptors, high selectivity for serotonin 5-HT2A receptors, and effects on GABA-ergic and glutamatergic circuitry have been implicated [25,26].

Conventional meta-analyses in medicine have almost exclusively focused on comparing mean effects across interventions. However, pharmacological treatment may not only affect the mean of a biological measure, but also the variance. Investigating how distinct interventions result in different variation of change in biological measures may provide valuable information regarding the nature of the interventions and study population. If clozapine's unique effects are related to the neurobiology of TRS,

one would expect a systematic difference in variation of symptom change following treatment with clozapine compared to other antipsychotics.

Objectives

In this study, we will test whether there is a systematic difference in variation of symptom change following treatment with clozapine compared to other antipsychotics by performing a meta-analysis of variance across previously reported double-blind randomised controlled trials. Additionally, to further investigate clozapine's uniqueness in relation to TRS, we will address whether clozapine's superiority extends to patients without operationally-defined TRS by undertaking a meta-analysis of mean difference.

Our main hypotheses are, 1) variation of symptom change would be smaller in TRS patients treated with clozapine compared to other antipsychotics, and 2) clozapine's superior efficacy compared to other antipsychotics would be limited to patients with TRS.

II. Methods

Protocol and registration

This protocol will be provided as supplementary material upon submission of the manuscript. The study will adhere to the Preferred Reporting Items for Systematic Reviews and Meta-analyses (PRISMA) statement [27], and will be registered with the PROSPERO international prospective register of systematic reviews.

Systematic Review

Eligibility criteria

Based on the PICOS reporting system, studies fulfilling the following characteristics will be identified.

Participants (P): Patients with schizophrenia and related psychoses
Intervention (I): Monotherapy with clozapine
Comparison (C): Monotherapy with any other antipsychotic medication
Outcomes (O): Change in psychopathology (total symptoms, positive symptoms, negative symptoms)
Study design (S): Double-blind randomised controlled trials (DBRCTs)

DBRCTs with both parallel and crossover design will be included. No specification will be applied for study duration. To be included, the primary publication of studies will have to be published in English. If several publications are found from the same authors based on overlapping participants, the publication with the largest sample, longest duration of intervention, and/or most detailed data regarding change in psychopathology will be selected.

Information sources

Published studies (including articles in press) from inception to February 2018 will be searched using Ovid (combining EMBASE, Medline, and PsycINFO), ClinicalTrials.gov, and the Cochrane Central Register of Controlled Trials (CENTRAL). Reference lists of relevant studies and review articles will be hand-searched to identify additional studies. The systematic search will be updated prior to submission.

Search

Two researchers will carry out the systematic search independently. No limits will be applied within the electronic search. The following search terms will be used for the combined Ovid search and Cochrane CENTRAL:

(clozapin* OR clozaril OR clopine OR denzapine OR fazaclo OR zaponex) AND (randomized controlled trial OR RCT OR controlled clinical trial OR double blind) AND (schizophr* OR schizoaffective OR psychosis)

Furthermore, the following search terms will be used for ClinicalTrials.gov:

Recruitment status: All studies
Condition or disease: (empty)

Other terms: clozapine
Country: (empty)

Study selection
Two researchers will assess the electronic records to identify studies meeting the eligibility criteria. Studies identified as eligible will be discussed between the two researchers to finalise the list of eligible studies. Any disagreements will be resolved through discussion including a third researcher. The PRISMA flow diagram will be used to summarise the process of study selection.

Data collection process
Two researchers will independently extract data from eligible studies using standardised spreadsheets. Any disagreements between the two researchers regarding extracted data will be resolved through discussion including a third researcher. If relevant, data may be extracted from related publications that refer to the same study. If data required for the meta-analysis is unreported (e.g. SD of change in total symptoms), corresponding authors will be contacted to request additional data.

Data items
For the meta-analysis of variance, the primary outcome measure will be standard deviation (SD) of change in total symptoms as measured by the Brief Psychiatric Rating Scale (BPRS) [28] or the Positive and Negative Syndrome Scale (PANSS) [29]. For the meta-analysis of mean difference, the primary outcome measure will be mean change in total BPRS/PANSS scores. Secondary outcome measures will include SD of change and mean change in positive and negative symptoms as measured by the BPRS/PANSS subscales, the Scale for the Assessment of Positive Symptoms (SAPS) [30], or the Scale for the Assessment of Negative Symptoms (SANS) [31].

The following variables will also be extracted: authors, year of publication, participant characteristics (age, duration of illness, treatment setting, definition of treatment resistance where applicable), study duration, parallel vs. crossover design, clozapine dose, name and dose of comparator, industry sponsorship, type of symptom scale used, and mean±SD total, positive and negative symptom scores at baseline and endpoint.

Outcome measures based on intention-to-treat (ITT) analysis will be prioritised when available.

Missing data
When SD of change in symptom scores is neither reported nor provided by the corresponding authors, reported standard errors and confidence intervals will be used to calculate these values if provided [32]. These calculated values, together with originally reported values and those provided by the corresponding authors, will be defined as “original values for SD of change in symptom scores”. Only original values for SD of change in symptom scores will be used in the meta-analysis of variance, as this is the primary outcome measure.

For the meta-analysis of mean difference, missing values for SD of change in symptom scores will be imputed based on the following steps outlined in the Cochrane Handbook for Systematic Reviews of Interventions [32].

1. Calculated using reported t values, P values, or F values
2. Calculated using mean correlation coefficients of studies reported in considerable detail, and applying this value to studies reporting mean±SD symptom scores at baseline and endpoint
3. Simple imputation of the mean SD of change calculated from other studies with similar design and characterisics

Study categorisation

Studies will be categorised into those strictly of TRS patients (TRS studies) and those that include patients not selected for treatment-resistance (non-TRS studies). These categories will reflect recommendations provided by the Treatment Response and Resistance in Psychosis (TRRIP) working group [7]. To be included in the TRS group, studies will be required to only include patients who were resistant to previous antipsychotic treatment. Studies which include patients with treatment-intolerance, relapse following non-adherence, or other non-treatment-resistant forms of schizophrenia will be defined as non-TRS studies.

TRS studies will be further assessed for the rigour with which TRS was assessed by determining the number of criteria required to define TRS that were met. The TRRIP consensus minimum criteria for TRS will be used for this purpose [7]. These specify a total of eight items, four regarding symptoms at the point of inclusion and four specifying the nature of failed adequate treatment trials. These criteria are summarised below.

<u>Symptoms at the point of inclusion</u>
Assessment: interview carried out using standardised rating scales
Severity: at least moderate symptom severity
Duration: symptom duration ≧12-weeks
Functioning: at least moderate functional impairment measured using validated scales

<u>Nature of failed adequate treatment trials</u>
Duration: ≧6-weeks
Dosage: ≧600mg/day chlorpromazine equivalent
Number of antipsychotics: at least two past antipsychotic trials
Adherence: ≧80% adherence based on at least two sources and antipsychotic plasma level monitoring

Criteria will be weighted equally and summed to determine the total number (maximum 8) that were used in assessment of TRS in a given study.

Risk of bias in individual studies

Two researchers will independently assess the risk of bias of individual studies using the Cochrane Collaboration's Tool for Assessing Risk of Bias [33]. Risk of bias will be assessed according to the categories shown below, with each graded as "Low Risk", "High Risk", or "Unclear Risk".

Selection Bias: Random Sequence Generation, Allocation Concealment
Performance Bias: Blinding of Participants and Personnel

Detection Bias: Blinding of Outcome Assessment
Attrition Bias: Incomplete Outcome Data Addressed
Reporting Bias: Selective Reporting
Other Bias

Any disagreements between the two researchers regarding risk of bias in individual studies will be resolved through discussion including a third researcher. The risk of bias of included studies will be summarized as part of the systematic review.

Meta-analysis

Summary measures

For the meta-analysis of variance, SD of change in symptom scores will be pooled across TRS and non-TRS studies to calculate the log variability ratio (lnVR) [34]. The lnVR represents the natural logarithm of the ratio of standard deviations for the experimental and control groups, as follows:

$$\ln VR = \ln\left(\frac{\hat{\sigma}_E}{\hat{\sigma}_C}\right) = \ln\left(\frac{s_E}{s_C}\right) + \frac{1}{2(n_E-1)} - \frac{1}{2(n_C-1)} \ ,$$

Where $\hat{\sigma}_E$ and $\hat{\sigma}_C$ are unbiased estimates of population SDs, s_E and s_C are reported sample SDs, and n_E and n_C are sample sizes for experimental (i.e. clozapine) and control (i.e. other antipsychotics) groups, respectively.

In biological systems, dependence between the mean and variance is common, in which larger mean values are associated with greater variance [35]. Therefore, a between-group difference in relative variability, as indexed with lnVR, may in part reflect a between-group difference in the mean. This mean-variance relationship is expected to be relevant when comparing the variability of symptom change between clozapine and other antipsychotics. Thus, we will calculate a complementary measure of relative variability which accounts for the difference in means, the log coefficient of variation ratio (lnCVR): [34]

$$\ln CVR = \ln\left(\frac{\hat{\sigma}_E/\bar{x}_E}{\hat{\sigma}_C/\bar{x}_C}\right) = \ln\left(\frac{s_E/\bar{x}_E}{s_C/\bar{x}_C}\right) + \frac{1}{2(n_E-1)} - \frac{1}{2(n_C-1)} \ ,$$

where $\bar{x}_E$ and $\bar{x}_C$ represent mean change for clozapine and other antipsychotics groups, respectively. The use of lnCVR to quantify group differences in variability is limited to ratio scale data [34], and is possible only in the case of data that has a true zero point. This is not the case for raw change scores which can be positive or negative. Thus, we will use converted $\bar{x}_E$ and $\bar{x}_C$ values in this equation, as follows:

$$\bar{x}_E = \left|\bar{x}_{E\,reported}\right| + (\bar{x}_{E\,baseline} - C) \ ,$$

$$\bar{x}_C = \left|\bar{x}_{C\,reported}\right| + (\bar{x}_{C\,baseline} - C) \ ,$$

where $\bar{x}_{E\,reported}$ and $\bar{x}_{C\,reported}$ are reported mean change, $\bar{x}_{E\,baseline}$ and $\bar{x}_{C\,baseline}$ are reported baseline scores, and C is the minimum score of the applicable rating scale (e.g. 30 for PANSS total) for clozapine and other antipsychotics groups, respectively.

For the meta-analysis of mean difference, we will calculate Hedges' g to quantify between-group differences in mean effects across TRS and non-TRS studies.

Synthesis of results
Meta-analyses will be performed in R 3.4.0 [36] using the metafor package [37]. We will require a minimum of three studies to conduct meta-analyses.

Primary outcomes relating to variance and mean difference will be pooled across studies using univariate random-effects models. Meta-analyses of secondary outcomes relating to positive and negative symptoms will be conducted in similar fashion. To assist interpretation of findings for the meta-analysis of variance, summary effect sizes for lnVR and lnCVR will be transformed back to a linear scale as follows:

$$VR = e^{lnVR} = \frac{\hat{\sigma}_E}{\hat{\sigma}_C} \ ,$$

$$CVR = e^{lnCVR} = \frac{\hat{\sigma}_E / \bar{x}_E}{\hat{\sigma}_C / \bar{x}_C} \ .$$

VR (or CVR) of 1 can be interpreted as equal variability in the clozapine and other antipsychotics groups, whereas a larger (or smaller) value would indicate greater (or less) variability in the clozapine group. For the meta-analysis of mean difference, standarised mean difference (SMD) will be used. Pooled effect sizes regarding VR, CVR, and SMD will be compared between TRS studies and non-TRS studies using a Wald-type test. All statistical tests will be carried out at a two-tailed alpha-level of 0.05.

Inconsistency between studies will be assessed using the I^2 statistics, with I^2 less than or equal to 50% indicating low to moderate inconsistency, whereas I^2 greater than 50% will indicate moderate to high inconsistency.

Risk of bias across studies
The likelihood of publication bias will be assessed by visual inspection of funnel plots and regression tests for funnel plot asymmetry.

Additional analyses
We will carry out a series of pre-specified sensitivity analyses: 1) excluding studies focusing on child and adolescence patients, 2) excluding studies with crossover design, and 3) excluding studies with imputed SD of change values for the meta-analysis of mean difference.

As pre-specified meta-regression, we will test the effects of the number of TRS criteria met, baseline symptom severity, clozapine dose, and duration of double-blind intervention as potential moderators on

variability and mean differences by using univariate mixed-effects meta-regression.

III. Time table / Work plan

Time schedule

We estimate a total of six months for the whole project. If necessary, the literature search may be repeated until the final manuscript is accepted by an academic journal. The date of the last literature search will be recorded.

Dissemination strategy

The results will be presented at international conferences, and ultimately published as a systematic review and meta-analysis in an international journal. In addition, essential scientific results will be made available to the public via the Institute of Psychiatry, Psychology & Neuroscience, King's College London website.

X. References

1 McGrath J, Saha S, Chant D, Welham J. Schizophrenia: a concise overview of incidence, prevalence, and mortality. Epidemiol Rev. 2008;30:67-76.

2 Whiteford HA, Degenhardt L, Rehm J, Baxter AJ, Ferrari AJ, Erskine HE, et al. Global burden of disease attributable to mental and substance use disorders: findings from the Global Burden of Disease Study 2010. Lancet. 2013;382(9904):1575-86.

3 Roick C, Heider D, Bebbington PE, Angermeyer MC, Azorin JM, Brugha TS, et al. Burden on caregivers of people with schizophrenia: comparison between Germany and Britain. Br J Psychiatry. 2007;190:333-8.

4 Agid O, Arenovich T, Sajeev G, Zipursky RB, Kapur S, Foussias G, et al. An algorithm-based approach to first-episode schizophrenia: response rates over 3 prospective antipsychotic trials with a retrospective data analysis. J Clin Psychiatry. 2011;72(11):1439-44.

5 Lieberman J, Jody D, Geisler S, Alvir J, Loebel A, Szymanski S, et al. Time course and biologic correlates of treatment response in first-episode schizophrenia. Arch Gen Psychiatry. 1993;50(5):369-76.

6 Conley RR, Buchanan RW. Evaluation of treatment-resistant schizophrenia. Schizophr Bull. 1997;23(4):663-74.

7 Howes OD, McCutcheon R, Agid O, de Bartolomeis A, van Beveren NJ, Birnbaum ML, et al. Treatment-Resistant Schizophrenia: Treatment Response and Resistance in Psychosis (TRRIP) Working Group Consensus Guidelines on Diagnosis and Terminology. Am J Psychiatry. 2017;174(3):216-29.

8 Kennedy JL, Altar CA, Taylor DL, Degtiar I, Hornberger JC. The social and economic burden of treatment-resistant schizophrenia: a systematic literature review. Int Clin Psychopharmacol. 2014;29(2):63-76.

9 National Institute for Health and Care Excellence. Psychosis and schizophrenia in adults: prevention and management. Clinical guideline 178. 2014. https://www.nice.org.uk/guidance/cg178.

10 Kane J, Honigfeld G, Singer J, Meltzer H. Clozapine for the treatment-resistant schizophrenic. A double-blind comparison with chlorpromazine. Arch Gen Psychiatry. 1988;45(9):789-96.

11 Lewis SW, Barnes TR, Davies L, Murray RM, Dunn G, Hayhurst KP, et al. Randomized controlled trial of effect of prescription of clozapine versus other second-generation antipsychotic drugs in resistant schizophrenia. Schizophr Bull. 2006;32(4):715-23.

12 McEvoy JP, Lieberman JA, Stroup TS, Davis SM, Meltzer HY, Rosenheck RA, et al. Effectiveness of clozapine versus olanzapine, quetiapine, and risperidone in patients with chronic schizophrenia who did not respond to prior atypical antipsychotic treatment. Am J Psychiatry. 2006;163(4):600-10.

13 Lieberman JA, Safferman AZ, Pollack S, Szymanski S, Johns C, Howard A, et al. Clinical effects of clozapine in chronic schizophrenia: response to treatment and predictors of outcome. Am J Psychiatry. 1994;151(12):1744-52.

14 Meltzer HY, Bastani B, Kwon KY, Ramirez LF, Burnett S, Sharpe J. A prospective study of clozapine in treatment-resistant schizophrenic patients. I. Preliminary report. Psychopharmacology (Berl). 1989;99 Suppl:S68-72.

15 Siskind D, Siskind V, Kisely S. Clozapine Response Rates among People with Treatment-Resistant

Schizophrenia: Data from a Systematic Review and Meta-Analysis. Can J Psychiatry. 2017;62(11):772-77.
16 Tiihonen J, Mittendorfer-Rutz E, Majak M, Mehtala J, Hoti F, Jedenius E, et al. Real-World Effectiveness of Antipsychotic Treatments in a Nationwide Cohort of 29823 Patients With Schizophrenia. JAMA Psychiatry. 2017;74(7):686-93.
17 Meltzer HY, Alphs L, Green AI, Altamura AC, Anand R, Bertoldi A, et al. Clozapine treatment for suicidality in schizophrenia: International Suicide Prevention Trial (InterSePT). Arch Gen Psychiatry. 2003;60(1):82-91.
18 Tiihonen J, Lonnqvist J, Wahlbeck K, Klaukka T, Niskanen L, Tanskanen A, et al. 11-year follow-up of mortality in patients with schizophrenia: a population-based cohort study (FIN11 study). Lancet. 2009;374(9690):620-7.
19 Siskind D, McCartney L, Goldschlager R, Kisely S. Clozapine v. first- and second-generation antipsychotics in treatment-refractory schizophrenia: systematic review and meta-analysis. Br J Psychiatry. 2016;209(5):385-92.
20 Samara MT, Dold M, Gianatsi M, Nikolakopoulou A, Helfer B, Salanti G, et al. Efficacy, Acceptability, and Tolerability of Antipsychotics in Treatment-Resistant Schizophrenia: A Network Meta-analysis. JAMA Psychiatry. 2016;73(3):199-210.
21 Howes OD, Kambeitz J, Kim E, Stahl D, Slifstein M, Abi-Dargham A, et al. The nature of dopamine dysfunction in schizophrenia and what this means for treatment. Arch Gen Psychiatry. 2012;69(8):776-86.
22 Howes OD, Egerton A, Allan V, McGuire P, Stokes P, Kapur S. Mechanisms underlying psychosis and antipsychotic treatment response in schizophrenia: insights from PET and SPECT imaging. Curr Pharm Des. 2009;15(22):2550-9.
23 Demjaha A, Murray RM, McGuire PK, Kapur S, Howes OD. Dopamine synthesis capacity in patients with treatment-resistant schizophrenia. Am J Psychiatry. 2012;169(11):1203-10.
24 Kim E, Howes OD, Veronese M, Beck K, Seo S, Park JW, et al. Presynaptic Dopamine Capacity in Patients with Treatment-Resistant Schizophrenia Taking Clozapine: An [(18)F]DOPA PET Study. Neuropsychopharmacology. 2017;42(4):941-50.
25 Seeman P. Clozapine, a fast-off-D2 antipsychotic. ACS Chem Neurosci. 2014;5(1):24-9.
26 Wenthur CJ, Lindsley CW. Classics in chemical neuroscience: clozapine. ACS Chem Neurosci. 2013;4(7):1018-25.
27 Moher D, Liberati A, Tetzlaff J, Altman DG, Group P. Preferred reporting items for systematic reviews and meta-analyses: the PRISMA statement. BMJ. 2009;339:b2535.
28 Overall JE, Gorham DR. The Brief Psychiatric Rating Scale. Psychological Reports. 1962;10:790-812.
29 Kay SR, Fiszbein A, Opler LA. The positive and negative syndrome scale (PANSS) for schizophrenia. Schizophr Bull. 1987;13(2):261-76.
30 Andreasen N. The Scale for the Assessment of Positive Symptoms (SAPS). University of Iowa: Iowa City; 1984.
31 Andreasen N. The Scale for the Assessment of Negative Symptoms (SANS). University of Iowa: Iowa City; 1983.
32 Higgins JPT, Green S. Cochrane Handbook for Systematic Reviews of Interventions Version 5.1.0. 2011. www.handbook.cochrane.org.
33 Higgins JP, Altman DG, Gotzsche PC, Juni P, Moher D, Oxman AD, et al. The Cochrane

Collaboration's tool for assessing risk of bias in randomised trials. BMJ. 2011;343:d5928.
34 Nakagawa S, Poulin R, Mengersen K, Reinhold K, Engqvist L, Lagisz M, et al. Meta-analysis of variation: ecological and evolutionary applications and beyond. Methods Ecol Evol. 2015;6(2):143-52.
35 Eisler Z, Bartos I, Kertesz J. Fluctuation scaling in complex systems: Taylor's law and beyond. Adv Phys. 2008;57(1):89-142.
36 R Core Team. R: A language and environment for statistical computing. 2017. https://www.R-project.org/. Accessed February 19, 2018.
37 Viechtbauer W. Conducting Meta-Analyses in R with the metafor Package. J Stat Softw. 2010;36(3):1-48.
